# Bias against Vitamin C in Mainstream Medicine: Examples from Trials of Vitamin C for Infections

**DOI:** 10.3390/life12010062

**Published:** 2022-01-03

**Authors:** Harri Hemilä, Elizabeth Chalker

**Affiliations:** 1 Department of Public Health, University of Helsinki, FI-00014 Helsinki, Finland; 2 Biological Data Science Institute, Australian National University, Canberra, ACT 2600, Australia; elizabeth.chalker@anu.edu.au

**Keywords:** attitude of health personnel, common cold, dietary supplements, evidence-based medicine, health care quality, access and evaluation, health knowledge, attitudes and practice, meta-analysis, micronutrients, quackery, respiratory tract infections

## Abstract

Evidence has shown unambiguously that, in certain contexts, vitamin C is effective against the common cold. However, in mainstream medicine, the views on vitamin C and infections have been determined by eminence-based medicine rather than evidence-based medicine. The rejection of the demonstrated benefits of vitamin C is largely explained by three papers published in 1975—two published in JAMA and one in the American Journal of Medicine—all of which have been standard citations in textbooks of medicine and nutrition and in nutritional recommendations. Two of the papers were authored by Thomas Chalmers, an influential expert in clinical trials, and the third was authored by Paul Meier, a famous medical statistician. In this paper, we summarize several flaws in the three papers. In addition, we describe problems with two recent randomized trial reports published in JAMA which were presented in a way that misled readers. We also discuss shortcomings in three recent JAMA editorials on vitamin C. While most of our examples are from JAMA, it is not the only journal with apparent bias against vitamin C, but it illustrates the general views in mainstream medicine. We also consider potential explanations for the widespread bias against vitamin C.

## 1. Introduction

In 1971, Linus Pauling published a meta-analysis of vitamin C and the common cold, which was one of the very first meta-analyses in medicine [1]. On the basis of results from four placebo-controlled trials with vitamin C doses >0.1 g/d, he calculated that it was highly unlikely that the decreases in the incidence of (*p* = 0.0014) and morbidity caused by the common cold (*p* = 0.000022) were caused by chance alone [1]. In another meta-analysis, Pauling focused on the two best trials that were available, and concluded that “the null hypothesis of equal effectiveness of ascorbic acid and a placebo is rejected at the level *p* less than 0.001” [2]. Pauling also wrote a book in which he argued that gram doses of vitamin C could prevent and alleviate colds [3].

Later randomized trials supported Pauling’s conclusions. In 2013, in our Cochrane review on vitamin C and the common cold, we calculated that regular vitamin C supplementation of ≥0.2 g/d shortened the duration of viral respiratory tract infections in adults by 7.7% (*p* = 0.00018) and in children by 14.2% (*p* = 0.000053) [4,5,6]. In five randomized trials with participants under heavy acute physical stress, vitamin C halved the incidence of common cold symptoms (*p* = 0.0000006) [4,7]. Furthermore, in British men who had particularly low dietary intake of vitamin C, supplementation decreased the recurrence of common cold infections during the follow-up period by 46% (*p* = 0.00005) [8,9].

Given such strong evidence that in certain contexts vitamin C is beneficial against colds, it is baffling that in mainstream medicine the effects of the vitamin against the common cold are largely ignored. In this review, we show that three papers from 1975 have been particularly influential in shaping the view of mainstream medicine against vitamin C. We describe the flaws in those papers and their influence on academic medicine. We also describe bias in the presentation of two recent vitamin C trial reports and in three editorials commenting on vitamin C research. The goal of this paper is to demonstrate the bias against vitamin C by using examples primarily from the trials on vitamin C for infections. Some reviews and studies on vitamin C and respiratory infections [10,11,12,13,14,15] have been flawed [16,17,18,19,20,21,22,23], but this paper does not systematically review the effect of vitamin C on infections, since this is covered in other publications [4,5,6,7,8,9,24,25,26,27,28,29,30,31,32].

## 2. The Karlowski (1975) Trial in JAMA

The primary report of the Karlowski trial was published in JAMA, which has very wide circulation and great impact in mainstream medicine [33,34]. In addition, the trial was carried out at the National Institutes of Health (NIH) and participants were employees of the NIH, which is a further factor that increased the influence of this particular trial. Furthermore, the principal investigator of the Karlowski trial was Thomas Chalmers, who was a particularly influential proponent of randomized controlled trials (RCTs) [35,36,37,38,39,40,41,42,43,44]. He was considered one of the “three individuals from an earlier generation [who] were particularly important in inspiring” the evidence-based medicine movement [45]. In one of his later texts, Chalmers commented on the Karlowski (1975) trial that “I am more proud of it than almost any other that I have published” [46]. Consequently, the Karlowski trial became, by far, the most influential trial of vitamin C for the common cold (Table 1 and Table 2).

The number of recorded common cold episodes was substantially smaller in the Karlowski trial than in three much larger trials that found significant benefit from vitamin C (Table 1). Nevertheless, on the basis of citations, the larger trials which demonstrated benefit from vitamin C have had much smaller influence in mainstream medicine than the Karlowski trial (Table 1). Paradoxically, the randomized placebo-controlled double-blind trial by Karlowski et al. also found a significant benefit from vitamin C, and therefore it is particularly perplexing as to why it has been used as evidence *against* the vitamin.

**Figure 1:**
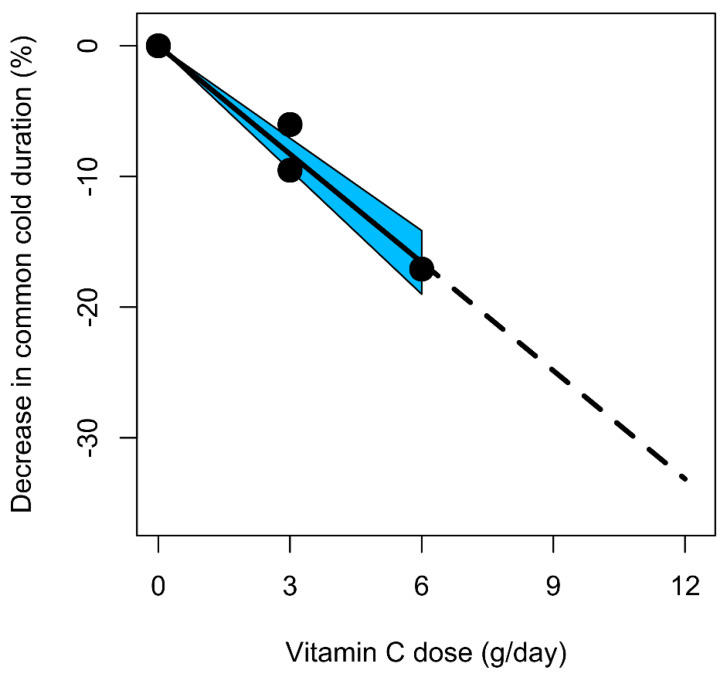
Dose–response relationship in the Karlowski (1975) trial. The mean durations in the four trial arms are plotted by vitamin C dose, with the placebo group serving as the base line. The test for slope gives *p* = 0.001 when forcing the regression line through the null effect, as defined by the placebo group. The blue area indicates the 95% CI for the regression line. The dashed line indicates the extrapolation to a dosage of 12 g/d, which ideally should have been tested in later trials because of the indication of a linear dose–response in the lower dose range. See Listing S2 for the calculation in the Supplementary Materials.

Karlowski randomized participants into four groups: (a) placebo, (b) 3 g/d of regular (prophylactic) vitamin C over the 9-month trial, (c) 3 g/d of vitamin C for five days of treatment if a participant had cold symptoms, and (d) a combination of 3 + 3 = 6 g/d of the regular and treatment vitamin C. The duration of colds in the 6 g/d group was 17% shorter than in the placebo group (*p* = 0.046), and the duration of colds in the two 3 g/d groups was between the placebo group and the 6 g/d group, consistent with linear dose dependency (Figure 1). In the JAMA report, Karlowski pointed out the apparent dose response and wrote: “each 3-gm increment of ascorbic acid would appear to shorten the mean duration of a cold by approximately half a day” [33]. However, Karlowski did not consider whether doses larger than 6 g/d might lead to an even greater benefit. Instead, the authors speculated that the observed benefit was caused by the placebo effect: “the effects demonstrated might be explained equally well by a break in the double blind” [33]. Thereby, the trial became a strong counterargument for vitamin C instead of serving as empirical evidence for the benefits of the vitamin [24] (pp. 21–27). Here, we first briefly describe the reasoning behind the placebo effect speculation, and then describe the flaws in the reasoning by Karlowski and Chalmers.

After the trial, it appeared that some of the participants had tasted their capsules, which may have undermined the validity of the trial, as blinding seemed to be broken. Karlowski and Chalmers reasoned that they could separate the participants who correctly guessed the contents of their capsules and those who guessed incorrectly. The benefit of vitamin C was limited to the group that guessed their capsules correctly. This led Karlowski to infer that tasting the capsules led to some participants being able to determine whether they were receiving vitamin C or placebo, and hence to the conclusion that the differences between the groups were caused by the placebo effect [33,34]. However, Karlowski’s placebo-effect proposal has numerous flaws [47,48,49,50]. The main problems are briefly described on the next page.

**Table 1:** Citations of major vitamin C common cold trials.

Trial [Refs] ^(1)^	Total No. of Episodes	Total Citations from 1997–2020 ^(2)^	Constituent of the Placebo	*p* for Testing Vitamin C Effect (2-t)
Karlowski (1975) [33]	249	109	Lactose	0.046
Lewis (1975) [34] ^(3)^		11		
Ludvigsson (1977) [51] ^(4)^	1279	20	not stated ^(4)^	0.016
Pitt (1979) [52] ^(5)^	1219	31	citric acid	0.023
Anderson (1972) [53] ^(6)^	1170	7	citric acid	0.001
Total excluding the Karlowski trial	3668	41		
Hemilä (1996) re-analysis [47] of the Karlowski (1975) trial		29		

^(1)^ All four trials were randomized, double-blind, and placebo-controlled. ^(2)^ Web of Science search 2021-11-5. Total number of citations is smaller than the sum for the 3 RCTs, because some citing papers cited 2 or 3 of the above vitamin C RCT reports. ^(3)^ The Lewis (1975) [34] is another report of the Karlowski (1975) [33] trial. It reports standard errors and other data not described in the primary JAMA report. ^(4)^ Ludvigsson trial [51]: Outcome: “Absence from school because of upper respiratory tract infection”: decrease of 14% in vitamin C group. “Every class was divided at random into two groups…In one of the groups the children received daily a fizzy tablet which contained 1000 mg vitamin C; in the other group the fizzy tablet looked and tasted the same but contained 10 mg [vitamin C] carried out totally double blind” (p. 91–92). ^(5)^ Pitt and Costrini trial [52]: Outcome: “Severity of colds”: decrease of 5.1% in vitamin C group. “… recruits were assigned randomly to either the vitamin C or placebo group from a list of consecutive numbers randomized in pairs…The vitamin C tablets each contained 500 mg of ascorbic acid in the anhydrous form, and the placebo tablets were formulated from citric acid and were indistinguishable in appearance and taste from the vitamin C tablets…Neither the recruits or drill instructors nor the physicians and corpsmen who treated the recruits were aware of which pill any individual recruit was taking” (p. 908). ^(6)^ Anderson trial [53]: Outcome: “Total number of days confined to house”: decrease of 30% in vitamin C group. “Each bottle of tablets was assigned a code number derived from a computer-generated list of consecutive numbers, randomized in pairs…Particular care was taken to ensure that the vitamin and placebo tablets were indistinguishable in appearance and taste. Pure ascorbic acid has a very strong and characteristic flavour which is difficult to imitate, and we therefore used a formulation containing 200 mg. of sodium ascorbate, 75 mg. of ascorbic acid and an artificial orange flavouring. The taste of this formulation was well matched by a placebo preparation containing 30 mg. of citric acid and the same orange flavouring and fillers. The effectiveness of the matching was established by asking 30 individuals to taste both tablets, and using pure ascorbic acid as reference, to judge which tablet contained the vitamin. Sixteen persons selected the placebo tablet and 14 the vitamin tablet. The effectiveness of the matching was verified at the end of the main study by the answers to the question ‘Do you think you have been on the vitamin or placebo tablet?’ Approximately half of the 818 subjects answered ‘Don’t know’, and the remainder were divided almost equally between those who guessed correctly and those who did not… After the bottles had been labelled the list of numbers was given for safe-keeping to a colleague who was not involved in the study…subjects were allocated to vitamin and placebo in a strictly double-blind randomized manner and the code was not broken until after all the data had been transferred to punch cards and initial tabulations carried out” (p. 504).

First, in their subgroup analysis by guessing, Karlowski gives the impression that all the participants were divided into two complementary groups: those who guessed correctly and those who guessed incorrectly [33]. However, 42% (105/249) of the recorded common cold episodes were missing from the subgroup analysis. Karlowski did not discuss these missing episodes. Paradoxically, the benefit of vitamin C was greater in this group than in the whole participant population [47], but this was not considered by Karlowski [33,34].

Second, Karlowski et al. ignored the fact that there were two kinds of capsules: regular and therapeutic. In their subgroup analysis (those who guessed correctly and those who did not), they did not describe which of the two capsules were guessed correctly or not [33]. There was no evidence that participants correctly guessed the contents of the therapeutic capsules, which were more effective, whereas there was evidence that participants correctly guessed the regular capsules, which were much less effective [47]. Such a finding is inconsistent with Karlowski’s speculation about the placebo effect as the explanation for the observed benefits of vitamin C.

Third, in vitamin C trials, the placebo for ascorbic acid has usually been citric acid (Table 1). Ascorbic acid and citric acid cannot be distinguished by taste. However, in the Karlowski trial, the placebo used was lactose, which is sweet [33]. Thus, in the Karlowski trial, a participant administered vitamin C would have had difficulty distinguishing whether their capsule contained ascorbic acid or citric acid, but a participant might infer they were taking the placebo because of the sweet taste of lactose. Surprisingly, more vitamin C participants guessed correctly than placebo participants [47]. This is also inconsistent with the placebo effect speculation.

Fourth, Karlowski did not properly consider other explanations for the ability of participants to correctly guess the contents of their capsules [33]. Subgroup analysis by a factor that is determined after a trial is initiated is highly dubious, since the factor may depend on the treatment. If a treatment is truly effective, the patient can infer the active treatment by the disappearance of symptoms [47,48,49]. As to vitamin C, the large double-blind trial by Pitt and Costrini used an indistinguishable citric acid placebo (Table 1), yet a statistically significant (*p* = 0.013) proportion of their participants were able to figure out their treatment correctly due to their subjective observations [24] (pp. 25–27). Thus, it is possible that participants taking vitamin C capsules in the Karlowski trial were able to correctly figure out the contents because of the physiological effects, rather than because of taste differences between the vitamin C and placebo capsules [47,48,49].

A re-analysis described the problems with the Karlowski–Chalmers placebo explanation [47]. Chalmers replied to the re-analysis, but the only error he pointed out in the re-analysis was the use of the term ‘tablet’, whereas the trial had used capsules [46,54]. So far, the 1996 re-analysis of the Karlowski trial [47] has not been challenged. However, the Karlowski trial continues to be extensively cited and remains highly influential, even after the 1996 re-analysis (Table 1 is restricted to citations after 1996).

Table 2 lists citations of the Karlowski trial by influential researchers. In addition, the Listing S1 in the Supplementary Materials presents extracts of papers that have cited the Karlowski trial as evidence of the lack of effect of vitamin C or as evidence of the placebo effect in action.

**Table 2:** Citations of the Karlowski (1975) trial by influential researchers and documents.

Source [Ref.]	Statement
Cecil Textbook of Medicine (1996, 2000, 2004) [55,56,57]	“a variety of actually ineffective treatments have been reported to be effective due to inadequate blinding of placebo recipients. One example of this phenomenon was a study of large dose of vitamin C to prevent colds, in which many placebo recipients dropped out of the study because they could tell by tasting the medication that they were not receiving the vitamin C [Karlowski 1975]”
Principles and Practice of Infectious Diseases (1979, 1985, 1990, 1995) [58,59,60,61]	“Many participants correctly surmised from the taste of the contents of the capsules used whether they were receiving vitamin C or a placebo (Karlowski 1975)”
Textbook of Pediatric Infectious Diseases (1987, 1992, 1998) [62,63,64]	“It is most probable that the reported benefits are a result of statistical artifacts and placebo effect due to poor study design rather than specific pharmacologic drug effects [Karlowski 1975]”
Recommended Dietary Allowances, 9th ed (1980) [65]	“Karlowski et al. (1975) found that when those subjects who had guessed the nature of their medication (ascorbic acid or placebo) were eliminated from consideration, the differences between the vitamin and placebo groups were not significant”
Evolution of Evidence for Selected Nutrient and Disease Relationships (2002) [66]	“Karlowski and colleagues (1975) conducted a small, double-blind study with 311 employees of the National Institutes of Health and concluded that vitamin C had ‘at best only a minor influence on the duration and severity of colds,’ and ‘the effects demonstrated might be explained equally well by a break in the double blind.’ ”
CONSORT statement [67]	“Unblinded outcome adjudicators may differentially assess subjective outcomes…These biases have been well documented” [Karlowski (1975) as one of the references]
Cochrane Handbook (1994, 2002, 2004, 2006) [68,69,70,71]	“Some research suggests that such blinding is important in protecting against bias (Karlowski 1975) …there is evidence that participants who are aware of their assignment status report more symptoms, leading to biased results (Karlowski 1975) ...Blinding is likely to be particularly important in research with subjective outcome measures such as pain ...(Karlowski 1975)”
Fundamentals of Clinical Trials (1982,1985,1998,2010) [72,73,74,75]	“A trial of the possible benefits of ascorbic acid in the common cold started out as a double-blind study (Karlowski 1975). However, it soon became apparent that many of the participants, most of whom were medical staff, discovered whether they were on ascorbic acid or placebo…Among those participants who claimed not to know the identity of the treatment, ascorbic acid showed no benefit over placebo. In contrast, among participants who knew or suspected what they were on, ascorbic acid did better than placebo. Therefore preconceived notions about the benefit of a treatment, coupled with a subjective response variable, may have yielded biased reporting.” “An evaluation such as that provided by Karlowski and colleagues for a trial of vitamin C is commendable”
Clinical Epidemiology (1986, 1996, 2006) [76,77,78]	“Lack of blinding…Because a subject’s suspicion of the group to which he or she had been signed so strongly influenced the results, and because a subject’s suspicion was much more often right than wrong, the validity of the vitamin C-placebo comparison was seriously compromised [in Karlowski 1975]”
Clinical and Translational Science: Principles of Human Research (2017) [79]	“Blinding (or masking) is essential in most explanatory trials...examples of incorrect results due to bias in trials without blinding (Karlowski et al., 1975)...reinforce the value of blinding”
Principles and Practice of Clinical Research (2007) [80]	“Blinding is essential in most explanatory trials since the opportunity for bias is substantial...Despite the rarity of deceit in clinical research, examples of incorrect results due to bias in trials without blinding (Karlowski 1975) …reinforce the value of blinding”
BMJ (1976) [81]	“American study of adult employees of the National Institutes of Health reported in 1975 found no significant prophylactic or therapeutic benefit from ascorbic acid”

## 3. Chalmers’ (1975) Review in American Journal of Medicine

In addition to the Karlowski trial, in 1975, Thomas Chalmers also published a meta-analysis of vitamin C and the common cold in the American Journal of Medicine [82]. That meta-analysis has been cited, for example, in textbooks of nutrition and infectious diseases and in nutritional recommendations, in which authors have claimed that vitamin C is ineffective against colds (Table 3) [24] (pp. 36–38). As one specific example of the significant influence of the Chalmers review, the Council on Scientific Affairs of the American Medical Association (AMA) officially stated in 1987 that “One of the most widely misused vitamins is ascorbic acid. There is no reliable evidence that large doses of ascorbic acid prevent colds or shorten their duration” [83] (p. 1934), which was based entirely on Chalmers’ 1975 review [82]. A review of early systematic reviews in medicine [84] listed the Chalmers review [82], but ignored Pauling’s two meta-analyses, though they were published four years earlier and, so far, their scientific validity has not been challenged [1,2]. One biography considered that the review on vitamin C and the common cold was one of the major papers by Thomas Chalmers [44,85].

Chalmers’ (1975) review has a number of serious flaws. The review was inconsistent in its inclusion/exclusion of trials and in selecting outcomes, and had data extraction and calculation errors [86]. In addition, Chalmers did not consider the doses of vitamin C used in the trials. In the extreme, Cowan et al. (1942) administered only 25 mg/d as their lowest supplementary dose [87], whereas Karlowski et al. (1975) administered 6000 mg/d of vitamin C as their higher dose [33]. This means a 240-fold difference between the doses. Based on the dose response as indicated in Figure 1, it is evident that a 25 mg/d dose is not relevant when testing the effects of vitamin C on common cold duration that Pauling proposed [1,2,3]. However, Chalmers presented these two trials side-by-side in his comparison table without mentioning the dramatic difference in the doses in the text or the table. 

**Table 3:** Major citations to the reviews by Chalmers (1975) and by Dykes and Meier (1975).

Source [Ref.]	Statement
Recommended Dietary Allowances, 10th ed (1989) [88]	“Several reviewers (Chalmers, 1975; Dykes and Meier, 1975) have concluded that any benefits of large doses of ascorbic acid for these conditions are too small to justify recommending routine intake of large amounts by the entire population”
Recommended Dietary Allowances, 9th ed (1980) [65]	“several reviewers (Chalmers, 1975; Dykes and Meier, 1975) believe that these benefits of large doses of ascorbic acid are too small to justify recommending routine intake of large amounts by the entire population”
American Medical Association (1987) [83]	“One of the most widely misused vitamins is ascorbic acid. There is no reliable evidence that large doses of ascorbic acid prevent colds or shorten their duration [Chalmers 1975]”
Principles and Practice of Infectious Diseases (1979, 1985, 1990, 1995) [58,59,60,61]	“Until truly effective and specific treatment becomes available, there will continue to be fads in the use of unproven remedies. The ingestion of large doses of vitamin C has been widely used as a preventive or therapeutic measure for colds. However, a careful analysis of the studies has indicated that a placebo effect could not be ruled out [Chalmers 1975]”
Human Nutrition and Dietetics, 10th ed (2000) [89]	“Chalmers (1975) carried out a similar analysis of 14 clinical trials and reported that severity of symptoms was significantly worse in patients who received the placebo. Unfortunately, many volunteers correctly guessed their treatment and when this was taken into account, differences in both the number and severity of colds were minor and insignificant”
Human Nutrition and Dietetics, 9th ed (1993) [90]	“... claiming that large daily doses of vitamin C reduced the likelihood of contracting the common cold. The popularity of this concept prompted at least 14 clinical trials, which failed to show an effect of vitamin C (Chalmers 1975)”
Nutrition, Concepts and Controversies, 2nd ed (1982), 6th ed (1994) [91,92]	“... in 1975 a physician [Chalmers] reviewed many of them. He found that, statistically, takers of vitamin C did indeed suffer fewer and milder colds than takers of placebos. The difference averaged … one tenth of one day per cold in favor of the vitamin C-takers”
Modern Nutrition in Health and Disease, 8th ed (1994) [93]	“The use of megadoses of vitamin C to prevent the common upper respiratory diseases remains an unproven claim. Fourteen studies have been reviewed of which eight were considered acceptable [Chalmers 1975]. Only minor and insignificant effects were noted in terms of the prophylactic benefit of administering megadoses of vitamin C”
New England Journal of Medicine (1980) [94]	“Other compounds, such as…vitamin C,…have been extensively studied in vitro and in vivo but have not been proved safe and effective antiviral agents [Chalmers (1975) and Dykes and Meier (1975)]”
Journal of the Royal Society of Medicine (2016) [95]	“[Chalmers (1975)] brought together 14 trials of ascorbic acid for the common cold and combined the results from eight of them…All differences in severity and duration were eliminated by analyzing only the data from those who did not know which drug they were taking”
Antimicrobial Agents and Chemotherapy (1988) [96]	“One clinical trial of ascorbic acid showed that the apparent benefit in the vitamin C recipients was accounted for by volunteers who had tasted the contents of their capsules and correctly identified the treatment. Reanalysis with omission of these subjects found no evidence of a treatment benefit [Chalmers (1975)]”

Given that Thomas Chalmers was the principal investigator for the Karlowski (1975) trial (see Section 2), Table 4 shows one of the most glaring errors in Chalmers’ (1975) review. In his main table, Chalmers stated that there were 89 participants in the placebo group and that the mean duration of colds in the placebo group was 6.3 days [82]. However, according to the Karlowski (1975) trial reports, the placebo was administered only to 46 participants, and the mean duration of colds in the placebo group was 7.14 days [33,34]. The erroneous 89 participants is the sum of 46 participants who were actually given placebo and 43 participants who were administered 3 g/d of vitamin C as a 5-day therapy. The erroneous duration of 6.3 days in Chalmers’ “placebo” group is the average of 5.92 days in the 3 + 3 g/d vitamin C group and the 6.71 days in the 3 g/d regular vitamin C group [33,34]. Thus, in Chalmers’ main table, half of the “placebo” group was given 3 g/d of vitamin C, and the “placebo” group’s common cold duration was the average for two vitamin C groups.

Correct data for the Karlowski trial are also shown in Table 4. Here the 3 + 3 g/d group is used as the vitamin C group, though more informative is the simultaneous analysis of all four groups by vitamin C dose (Figure 1). Further flaws in Chalmers’ review have been described previously in great detail [86].

In his main table, Chalmers calculated that common cold episodes were 0.11 ± 0.24 (SE) days shorter in the vitamin C groups [82]. Even if real, a 0.11 day (= 2.6 h) decrease in common cold duration would have no clinical relevance, and the large SE indicates that the slight difference is explained by random variation. Therefore, Chalmers concluded that there was no valid evidence to indicate that vitamin C is beneficial in the treatment of the common cold. 

However, a re-analysis of the trials known to Chalmers in 1975, which had used ≥1 g/d of vitamin C, i.e., trials that tested Pauling’s proposal [1,2,3], showed that colds were 0.93 ± 0.22 (SE) (*p* = 0.01) days shorter in the vitamin C groups [86]. Thus, an estimate more than eight times higher than Chalmers’ estimate was obtained by using the correct values and restricting the analysis to trials which used doses as high as Pauling had suggested. Furthermore, Chalmers calculated the effect of vitamin C on the absolute scale, i.e., as reduction in duration of the common cold in days. However, the duration of colds in the control groups varies substantially, and therefore the relative scale is much more useful in the analysis of such an outcome [86,97,98,99]. Had Chalmers used the relative scale, he would have found even stronger evidence that vitamin C shortens common cold duration by 21% ± 3% (SE) (*p* = 0.001) [86]. Finally, given the strong indications of dose dependency in the Karlowski trial (Figure 1), Chalmers could have restricted his analysis to trials that administered ≥2 g/d vitamin C, and combining their *p*-values would have led to *p* = 0.000002 [100]. Thus, using correct data from trial reports and appropriate statistical methods, a review in 1975 could have concluded that there was very strong evidence that vitamin C differs from placebo in its effects on the common cold.

## 4. Dykes and Meier (1975) Review in JAMA

In 1975, JAMA published the Dykes and Meier (1975) review on vitamin C and the common cold [101]. The second author of the review, Paul Meier, was a famous medical statistician, the second author of the widely popular Kaplan–Meier method [102,103,104]. Consequently, this review on vitamin C in a widely circulated journal became highly influential. It has also been a standard reference in textbooks on nutrition and infectious diseases, and in nutritional recommendations, when justifying the claim that vitamin C has no effects on the common cold (Table 3) [24] (pp. 42–45). Again, there are several shortcomings in the review [100]. 

Dykes and Meier justified their review by referring to Pauling’s meta-analysis [1]. They stated that “Pauling gave great weight to the 1961 study of schoolchildren in a skiing camp in Swiss Alps by Ritzel”. It then seems illogical that Dykes and Meier did not highlight the results of the Ritzel trial in their paper. They failed to mention that in the Ritzel trial, there was a 45% decrease in the incidence of colds (*p* < 0.03) [1] and a 61% decrease in the total number of days of colds in the group administered 1 g/d of vitamin C [105]. Hiding such quantitative findings from the readers of JAMA was misleading. Concealing the actual results prevented critically minded readers from drawing their own conclusions about the Ritzel trial, which was randomized, double-blind, and placebo-controlled [105,106]. The Ritzel (1961) trial falls in the small group of trials that found significant preventive effects of vitamin C in participants under heavy short-term physical stress [4,7], and thereby it seems to reflect a genuine biological effect instead of being a statistical artifact.

Dykes and Meier discussed the results of the Anderson (1972) trial [53], but did not present any estimates of effect, which again prevented the reader from considering the findings. Dykes and Meier wrote that “the estimated effect is considerably less than that predicted by Pauling for the dose level”. Anderson had reported a 30% decrease (*p* = 0.001) in the ‘total number of days confined to house’ per participant (Table 1). Dykes and Meier’s comment seems irrelevant, as many readers might consider that a 30% effect with an inexpensive nutrient which is safe in the doses that were used would justify and encourage further research.

Dykes and Meier (1975) commented on the Coulehan et al. trial (1974) [107] with Navajo schoolchildren that “Because the data required for an appropriate analysis are not presented, the statistical significance of the differences reported cannot be considered to have been established” [101]. In fact, Coulehan et al. (1974) reported, in their table 4, the number of school children who were ‘never ill on active surveillance’ (Table 5). Thus, data required for an appropriate analysis were presented and could have been re-analyzed by a statistically oriented reviewer. The claim that significant differences “cannot be considered to have been established” was greatly misleading.

Although there were numerous flaws in Karlowski’s trial interpretation, as described in Section 2, Dykes and Meier uncritically accepted the placebo-effect explanation for the results of the trial. 

Pauling was critical of the Dykes and Meier review and considered that “some significant studies in this field were not mentioned by Dykes and Meier, and some important aspects of the studies discussed by them were also not mentioned by them” [108]. Pauling thus wrote a manuscript in which he described the results of the relevant trials, his own interpretations of the findings, and criticism of Dykes and Meier’s arguments. Pauling submitted his manuscript to JAMA, but his paper was rejected, even after Pauling twice made revisions to meet the suggestions of the referees [108,109,110]. The manuscript was finally published in a minor journal [108,111]. The rejection of Pauling’s review by JAMA seems unethical, since its readers were thereby prevented from seeing the other side of an important scientific controversy about an intervention that might have substantial public health relevance.

## 5. Consequences of the Three 1975 Papers

The Karlowski trial and the Dykes and Meier review were published in the same issue of the highly influential JAMA, on 10 March 1975. Thus, it must have been very convincing to the reader of JAMA that vitamin C is useless for the common cold, because of the simultaneous publication of a review by a top statistician and a new randomized trial carried out at the NIH by a top clinical trialist. Furthermore, the trial appeared to also explain away the previously claimed benefits of the vitamin, as the placebo effect was presented as the evident explanation: “there is strong evidence in our data that the effects of ascorbic acid on duration of colds and severity of symptoms are the result of suggestion” [34]. Many other trials had used a valid placebo (Table 1), but that was not mentioned in the Karlowski trial reports. The implication of the placebo effect as a universal explanation for the positive findings in earlier vitamin C trials might have been less convincing had the strengths of many earlier RCTs been properly discussed. The two papers have been standard references in textbooks of medicine and nutrition, and in nutritional recommendations, when claiming that vitamin C has no effect on the common cold (Table 2 and Table 3).

In addition to being cited in the medical and nutrition contexts as evidence that vitamin C is ineffective on the common cold, the Karlowski (1975) trial has also been extensively cited in clinical trial textbooks and other literature as evidence of the placebo effect in action (Table 2). Over four decades, the popular textbook *Fundamentals of Clinical Trials* has cited the Karlowski trial, with the latest edition dedicating a whole paragraph to describing the placebo effect explanation of the trial. Two of the later editions [74,75] cited the trial, ignoring that the original analysis was shown to be erroneous in 1996 [47]. 

Similarly, over three decades, the textbook *Clinical Epidemiology* cited the Karlowski trial, presenting a separate paragraph as an example of “one study in which the identical appearance of drug and placebo was achieved but blinding was not is instructive to review here” [78]. The Cochrane Collaboration is a major organization promoting evidence-based medicine, yet in several editions of their handbook they also have cited the Karlowski trial as evidence of the placebo effect, despite the original analysis of the trial being shown to be erroneous [69,70,71]. 

Furthermore, Robert Califf, the Commissioner of the US Food and Drug Administration (FDA) from 2016 to 2017 and again since 2021, has cited the Karlowski trial in book chapters on clinical trials, using the trial as evidence of the placebo effect [79,80] (Table 2). Thus, the Karlowski trial is a zombie that does not die, even though the errors are well documented [24,47,48,49,50,54]. See Listing S1 in the Supplementary Materials for extracts of citations to the Karlowski paper.

This widespread use of the Karlowski trial as an example of the placebo-effect in action in clinical trial textbooks and book chapters is particularly unfortunate, since in addition to physicians and nutritionists, it gives the impression to methodologically oriented people that the effects of vitamin C are just placebo effects. Thus, when methodologically oriented researchers comment on the effects of vitamin C, they can also be biased. It is likely that this background explains, in part, the flaws in five recent papers, which are discussed in the following sections.

The third 1975 paper was published in the American Journal of Medicine, which is not as prestigious as JAMA, yet having Thomas Chalmers as its author ensured that that paper was also highly influential. Therefore, it too became a standard reference in textbooks and in reviews on vitamin C and on respiratory infections (Table 3). 

**Figure 2:**
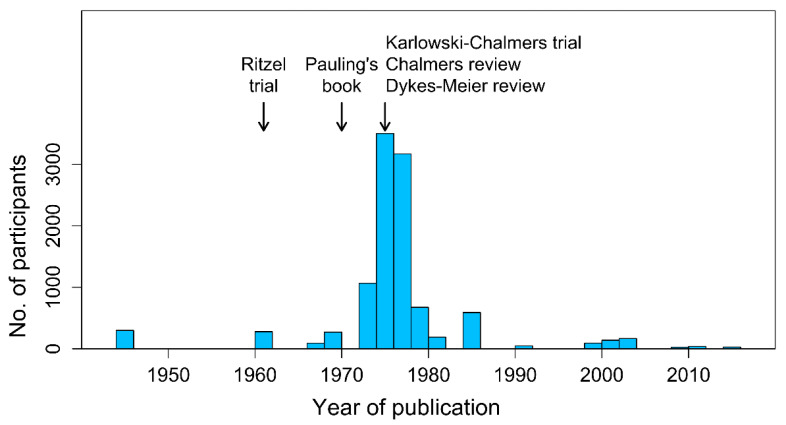
The numbers of participants over time in placebo-controlled trials for which ≥1 g/d of vitamin C was administered. The numbers of participants in studies published over two consecutive years are combined and plotted for the first of the two years. This figure is based on data collected by Hemilä and Chalker (2013) [4,5]. The randomized double-blind placebo controlled trial by Ritzel (1961) [105,106] had great influence on Pauling’s conclusions on vitamin C and the common cold [1,2,3]. Pauling’s book [3] led to intensive research on vitamin C and the common cold, but the interest vanished after the publication of the three papers in 1975 [33,82,101]. An earlier version of this figure has been published previously [32].

Pauling’s 1970 book [3] and other activities caused wide-spread interest in vitamin C in the early 1970s, both among lay people and among academic researchers [24,32,112]. As a consequence of Pauling’s book, 29 placebo-controlled studies were published in the eight-year period from 1972 to 1979 [4] (Figure 2). The total number of participants in those trials was 8409, which corresponds to an average of 290 participants per study. After the mid-1970s, interest in vitamin C and the common cold plummeted within a few years. During the 30-year period from 1985 to 2014, only 11 placebo-controlled trials comprising just 538 participants in total were published, with a mean of 49 participants per study [4,32]. Thus, the number of trials declined from the rate of 3.6/year to 0.37/year, but also, their average size declined by 83%. This rapid decline of interest in vitamin C can be attributed largely to the three flawed papers from the year 1975, as they led to increased bias against vitamin C [24,32,112] (Table 2 and Table 3). 

This lack of interest in the effects of vitamin C on the common cold and other infections is very unfortunate when we consider the statistically highly significant effects observed in the controlled trials on vitamin C (see Section 1 and Table 1). Although the regular supplementation trials serve as proof of the concept that vitamin C can shorten the duration of colds and alleviate severity [4,24,25,26,27,31], there are many relevant questions that remain unanswered.

For example, how do the findings on regular supplementation of healthy participants translate to treatment that starts after the onset of symptoms? So far, the therapeutic trials have not demonstrated a consistent benefit of vitamin C, but as we pointed out previously, “technically the therapeutic trials are in several ways much more complicated than regular supplementation trials. If the timing of supplementation initiation, the duration of supplementation, or the dosage, influence the size of the benefit, false negative findings might result from inappropriate study protocols” [4]. Four therapeutic trials used short two to three-day vitamin C administration, and the doses have been small when compared with the maximum dosage of 6 g/d in the Karlowski (1975) trial, and even smaller when compared with the extrapolation to the possible benefits of 12 g/d (Figure 1).

Despite the indication of dose dependency (Figure 1) [4,26,47], we still do not know what the optimum vitamin C doses are or how great the benefits of those doses would be. In addition, given the observed variations in the effects of vitamin C between certain population subgroups [4,8,24,27,32,53], we do not know who would benefit most. It is frustrating that such questions still remain unanswered 50 years after Pauling’s meta-analyses [1,2]. It is likely that our lack of answers to these questions is largely due to the three papers from 1975 (Figure 2).

## 6. CITRIS-ALI (2019) Trial in JAMA

Animal studies have indicated that vitamin C may have an effect on sepsis [113,114]. The CITRIS-ALI trial was carried out to test such a hypothesis in humans, and it was published in JAMA [115]. The trial abstract reported that there were no significant differences between the vitamin C and placebo groups in the primary end point (modified Sequential Organ Failure Assessment score [SOFA]). The abstract failed to mention that in the vitamin C group there were statistically significant benefits of vitamin C for three out of four clinically relevant outcomes that were measured. One of them was mortality, which is a clinically much more relevant outcome than organ failure scores.

In the discussion section, the authors wrote [115]: “Among the 46 secondary outcomes that were examined in this trial, 43 showed no significant differences between the vitamin C group and the placebo group, although vitamin C compared with placebo was associated with a significant reduction in 28-day all-cause mortality, and with significantly increased ICU-free days to day 28 and hospital-free days to day 60. However, these findings were based on analyses that did not account for multiple comparisons and therefore must be considered exploratory.” This is misleading [116].

First, the CITRIS-ALI trial registration listed just 18 secondary outcomes [117]. Many of them were measured at several time points, but that does not account for 46 independent secondary outcomes. If there is no effect on a biomarker, it is likely that the lack of effect is uniform over time. Counting the measurements at several time points as if they were independent observations makes the multiple comparison issue appear much greater than it is.

Second, a researcher interested in the effects of vitamin C on mortality can legitimately deny interest in the biomarkers. Biomarkers are used in research because they correlate with clinically relevant outcomes; however, there are several examples when effects on surrogates have diverged from effects on clinically relevant outcomes, and therefore their use is often discouraged [118,119,120,121]. Furthermore, when there are data available on clinically relevant outcomes, focus on the biomarkers is not logical, as the former are of primary interest whereas the biomarkers are just correlates. Four outcomes in the CITRIS-ALI trial were clinically relevant [115]. The significant effect on three of them is a much more relevant finding than the lack of effects on biomarkers.

Third, prior research is relevant for justifying research questions and interpreting findings, but was not properly discussed in the CITRIS-ALI report [115]. Given that, in earlier studies, vitamin C was reported to shorten ICU and hospital stay [122,123] and decrease mortality [124], research on these clinically relevant outcomes is not a fishing expedition without any biological rationale, but testing well-justified hypotheses.

Fourth, the intention-to-treat (ITT) principle is very important in RCTs [125,126,127]. It means that all participants should be included in the analysis, and they should be analyzed in the group to which they were allocated. However, the primary statistical analysis of the CITRIS-ALI trial excluded participants who died [115]. Such an exclusion is inappropriate, as it causes serious bias in the analysis of the trial findings [128]. In fact, when the maximum SOFA scores were imputed for the participants who had died, the effect of vitamin C at 96 h was statistically significant, with *p* = 0.03 [129]. Thus, the *p* = 0.86 for the difference between vitamin C and placebo groups at 96 h published in the original trial report [115] was based on a flawed statistical approach, as it did not follow the ITT principle. 

**Figure 3:**
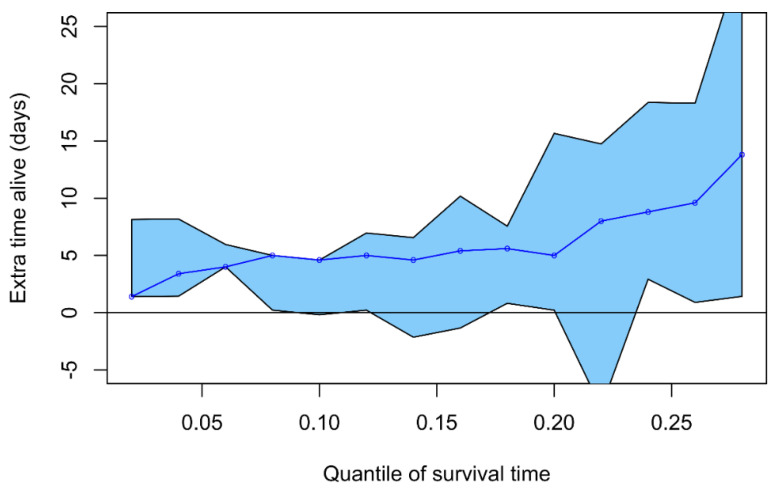
The QTE analysis of the CITRIS-ALI trial. The horizontal axis shows the quantile of survival. The vertical axis shows the difference in the life span between the vitamin C and placebo groups on the same quantile. The blue line shows the QTE estimate and the shaded region shows its 95% CI. This QTE analysis was carried out with the *cqr* program of the *quantreg* package of R [131]. The extraction of the CITRIS-ALI trial data has been described previously [116]. See Listing S4 for the calculation in the Supplementary Materials.

The most important clinically relevant outcome in the CITRIS-ALI trial was mortality, and measurement of a dozen biomarkers should not distract focus away from the mortality findings. Furthermore, the published survival curves show that the effect of vitamin C is not uniform over the follow-up period [115]. We calculated that, during the period up to day four, vitamin C decreased mortality by 81% (95% CI 45–94%) but had no effects thereafter, with the two-period model improving the Cox regression when compared with no vitamin C effect (*p* = 0.002) [116]. In the CITRIS-ALI trial, vitamin C was administered for four days, and thus there is a close association between the vitamin administration period and the period of benefit. Another approach to analyzing the data is the use of quantile treatment effects (QTE). For example, the 0.25 quantile (25th percentile) corresponds to 5 days alive in the placebo group and 13 days alive in the vitamin C group, and thereby the length of life was 8 days longer in the vitamin C group (Figure 3). For several segments in the QTE analysis, the 95% confidence interval does not include zero, which indicates that there was a difference in survival between the vitamin C and placebo groups.

The misleading reporting of the CITRIS-ALI trial is puzzling. It may have arisen from journal reviewers and/or editors dictating what they are willing to publish or not, or from poor judgement by the authors. We tend to agree with the view of Marik and Payen that it is likely the editor and/or reviewers went to great lengths to require the authors to emphasise that the mortality benefit was one of many secondary endpoints, which could have been significant purely by the play of chance. Furthermore, as the primary endpoint was negative, this must be a negative study [130]. As described above, such reasoning is not sound. Thus, this case is very similar to the Karlowski (1975) trial described in Section 2. Both trials were positive on a clinically relevant outcome, but in both cases, JAMA published a version in which the positive finding from vitamin C was camouflaged by the placebo-effect speculation [33] or the multiple-comparison problem [115]. If Marik and Payen are correct, one may wonder why the authors decided to publish a misleading version of their trial in the highly influential journal, when it would have been possible to publish an objective and statistically more thorough version in a less prestigious journal.

## 7. COVID-A to Z (2020) Trial in JAMA Network Open

There are various lines of reasoning to indicate that vitamin C may have an effect on COVID-19 [132]. The COVID A to Z trial was carried out to test whether 8 g/d of vitamin C might increase the rate of recovery of COVID-19 outpatients [133]. The trial has serious methodological shortcomings [134].

The intention in the COVID A to Z trial was to recruit 520 patients. However, the trial was “stopped early for futility” after recruitment of only 214 patients [133]. Stopping early was inappropriate. 

In the methods section, the authors assumed a 1.0-day difference in the primary outcome, which was “time to a 50% reduction in symptom severity between the usual care arm and one of the intervention arms” [133]. This indicates that a 1.0-day reduction was considered clinically important by the authors; otherwise, they would not have used such an assumption in their sample size calculation. 

The trial results were reported as follows: “Patients who received usual care without supplementation achieved a 50% reduction in symptoms in a mean (SD) of 6.7 (4.4) days compared with a mean (SD) of 5.5 (3.7) days for patients receiving ascorbic acid” [133].

Thus, the primary outcome—time to a 50% reduction in symptom severity—was 1.2 days shorter in the vitamin C arm than the usual care arm. This means that the observed effect was 20% greater than the effect assumed in the sample size calculation (1.2 vs. 1.0). It is illogical to stop a trial early because of “futility” when the observed effect is greater than the expected effect. The authors did not discuss this paradox in their paper.

Another serious shortcoming of the trial was the lack of placebo. Experiencing virus symptoms is subjective, and if there is no blinding there can be systematic bias between the groups. The bias can be in either direction. If a patient believes that vitamin C is effective, he or she might underreport symptoms. On the other hand, when patients are given an active intervention, such as vitamin C, they may observe and report symptoms at a lower threshold compared with the usual care arm. In the context of a controlled trial, placebo tablets are inexpensive, yet the authors do not justify why none were used [133]. Furthermore, the authors are not explicit with what they mean by an “open label trial”, that is, whether the patients were actually told what they were receiving and how that was communicated. The psychological effects can be quite different depending on the wording used to describe the intervention. Further methodological problems are described elsewhere [134].

In addition to the methodological flaws, the statistical analysis of the COVID A to Z trial was also inappropriate. Several patients did not recover by the end of the 28-day follow-up. In statistics, such patients are classified as ‘censored’. When there are censored observations, it is not appropriate to calculate the mean, since we do not know when the censored patients were actually cured. The day of censoring should not be used as the day of recovery. However, in the COVID A to Z trial, the 6.7 day mean duration in the usual care group was based on imputing 28 days as the day of recovery for the six censored patients.

**Figure 4:**
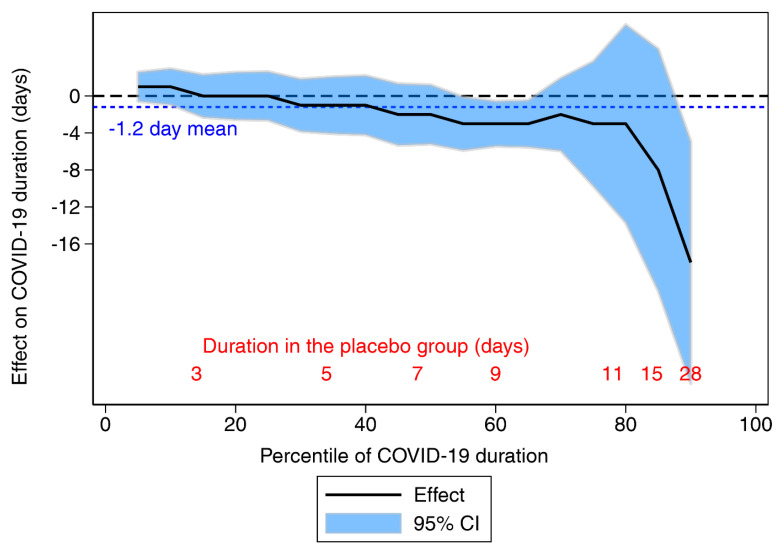
The QTE analysis of the COVID A to Z trial. The horizontal axis shows the percentile of COVID-19 duration. The vertical axis shows the difference in the duration in the vitamin C and usual care arms on the same percentile. The continuous black line indicates the QTE of vitamin C and the shaded region indicates its 95% CI. The horizontal black dashed line indicates the null effect. The blue dotted line shows the 1.2-day mean effect reported by Thomas et al. [133]. The red figures at the bottom indicate the lowest percentile level for the indicated COVID-19 duration in the placebo group. This QTE analysis was carried out with the *sqreg* program of STATA. The extraction of the COVID A to Z trial data has been described previously [134]. See Listing S5 for the calculation in the Supplementary Materials.

A statistically appropriate approach to analyzing data with censored observations is, for example, one using Cox regression. We calculated that vitamin C administration increased the recovery rate of COVID-19 patients by 70% (95% CI 6.8% to 170%, *p* = 0.025) during the 28-day follow-up [134]. Furthermore, analysis of the QTE indicated that there was no evidence of benefit for patients who suffered from COVID-19 for less than a week, whereas four-week COVID-19 symptoms might be shortened by about two weeks, with the 95% confidence interval being far from the null effect line (Figure 4).

It is baffling that the authors started the trial without a placebo, and that they stopped the trial early when the results were more positive than expected. In the trial report, one of the authors, Carla McWilliams, “reported receiving consulting fees from Gilead Sciences outside the submitted work” [133]. In another recent report, the related phrase was “Dr. Carla McWilliams reports being a paid consultant for Gilead” [135]. Gilead Sciences is the maker of remdesivir, which was the first treatment for COVID-19 approved by the FDA [136]. This seems a particularly serious conflict of interest, but JAMA seems to have ignored it. What motivation does such a researcher have to find out whether a very cheap nutrient is actually effective against COVID-19 when an expensive drug is available from a company for which she is a consultant?

Given the lack of placebo, and early termination because of ‘futility’ when the trial found an effect greater than expected, it is puzzling that such a paper was published without expert critical review. In our view, it seems highly unlikely that a trial which is methodologically flawed, such as the COVID A to Z trial, would have been published if the findings were presented positively in the manuscript, arguing that vitamin C was beneficial with *p* = 0.025. In such a case, it seems highly likely that JAMA reviewers and editors would have pointed out that a trial being stopped early without good reason is dubious, and a lack of placebo indicates that any differences could be explained by the placebo effect.

## 8. Editorial on Vitamin C in JAMA by Brant and Angus

An editorial on the CITRIS-ALI trial report was published in JAMA [137], which was misleading on several issues [138]. Brant and Angus wrote that “Although unproven, common folklore holds that [vitamin C containing] orange juice and lemons speed recovery from both influenza and the common cold, apparently due to a boost to the immune system” [137]. Factual statements in scientific papers are expected to be supported by evidence, but no references were supplied for this statement. It seems that Brant and Angus did not undertake a literature search before writing their editorial, otherwise they could not have made such a strong statement.

As described in Section 1, Pauling showed that by 1970 there was strong evidence that vitamin C has a beneficial effect on the common cold, and further placebo-controlled trials have demonstrated that, in some contexts, vitamin C can affect common cold incidence and duration. This large body of research was ignored by Brant and Angus.

In the editorial [137], Brant and Angus also wrote that “Linus Pauling, winner of 2 Nobel prizes, claimed a beneficial role for vitamin C across a wide range of diseases, including terminal cancer [139]”. This statement was based on one reference. However, that reference does not once mention cancer, and it is a paper discussing the evolutionary approach to determining ‘natural’ vitamin C intake levels when considering human ancestry [139]. Pauling published over a dozen articles on vitamin C and cancer, e.g., [140,141,142,143,144,145], but none of them were mentioned by Brant and Angus. Thus, by giving an incorrect reference [139] to their statement, Brant and Angus gave the impression that Pauling did not write any scientific papers on vitamin C and cancer.

Brant and Angus wrote about the CITRIS-ALI trial: “The authors also evaluated 43 secondary outcomes, including a variety of measures of inflammation and organ recovery”. However, as noted in Section 6, the study protocol of the CITRIS-ALI trial had only 18 secondary outcomes, not 43 [117]. Brant and Angus counted numerous time points for the 18 secondary outcomes as if they were independent observations. Furthermore, in evidence-based medicine we are interested in clinically relevant outcomes, and not in surrogates such as biomarkers. When there are data available on clinically relevant outcomes, surrogates are not relevant. In the CITRIS-ALI trial [115], there were only four clinically relevant outcomes, and the primary focus should be on them [116].

Finally, Brant and Angus did not critically appraise the statistical analysis of survival in the CITRIS-ALI trial. They write “28-day all-cause mortality rates…statistically significant without adjusting for multiple comparisons (*p* = 0.01)” [137]; however, visually, it is evident that the ratio of the survival curves, on which the *p* = 0.01 is based, is not constant over time [115]. This was confirmed statistically, as the two-period Cox regression model is significantly better than the uniform model (*p* = 0.006 for testing the superiority of the two-period model compared with a uniform vitamin C effect) [116]. Furthermore, compared to the model with no vitamin C effect over the 28-day period, the two-period model improves the Cox regression with *p* = 0.002. Thus, the *p* = 0.01 shown by Brant and Angus gives a misleading impression of the differences between the vitamin C and placebo curves in the CITRIS-ALI trial. The differences are much less likely to be explained by chance [116].

## 9. Editorial on Vitamin C in JAMA by Michos and Cainzos-Achirica

There was also an editorial published on the COVID A to Z trial report [146] which was misleading on several issues [147].

Michos and Cainzos-Achirica write [146] that “the evidence supporting supplements as a treatment for viral infections is rather limited [148]”. However, the reference used to support that statement is a superficial discussion of numerous supplements, in which the text specifically about vitamin C was just 145 words long [148]. Michos and Cainzos-Achirica ignored the Cochrane review on vitamin C and the common cold, which is 104 pages long [4], and a review on vitamin C and infections in general that had 161 references [32]. These latter reviews are much more informative sources on vitamin C and viral infections.

Michos and Cainzos-Achirica further write about the COVID A to Z Trial that “after an interim analysis, the safety monitoring board recommended the study be stopped early for futility after enrollment of only 214 participants because of the low probability of detecting significant differences between the study groups in terms of the primary end point” [146]. As pointed out in Section 7, the difference between the vitamin C and usual care arms found an effect greater than expected, and thus it was illogical to stop such a trial early for futility. Michos and Cainzos-Achirica did not consider this paradox. How is it possible that a 1.0 day reduction in duration was clinically important enough to justify the trial, but the observed 1.2 day reduction was futile and justified early stopping?

In the COVID A to Z trial, interventions were not blinded and no placebo was used in the control group. Michos and Cainzos-Achirica speculate [146] that “this could have exaggerated the potential benefit of the interventions”. However, it is also possible that the lack of placebo may lead to an underestimation of the effect. When a patient is given an active intervention, it is possible that he or she tends to observe and report symptoms more sensitively compared with the “usual care” group. We do not know the direction of the bias.

Michos and Cainzos-Achirica further comment that “more adverse effects (nausea, diarrhea, and stomach cramps) were reported in the supplement groups than in the usual care group” [146]. Here, they ignore vitamin C pharmacokinetics. The COVID A to Z trial report described doses of “8000 mg of ascorbic acid (to be divided over 2-3 times per day with meals)” [133].

Absorption of vitamin C is saturated with high doses [149]. Therefore, it seems evident that a large single dose is likely to cause more stomach ailments than the same daily dose divided into many small and frequent doses over the day. For example, in the large trial by Anderson et al. in 1974, the instruction for the 8 g dose per day was “16 tablets (two every hour) on the first day of any illness”, i.e., eight doses over a day [150]. The COVID A to Z trial report does not justify the choice of two or three doses over the day [133], which can cause greater intestinal discomfort and falsely suggest greater adverse effects of vitamin C compared with a more rational administration. Thus, Michos and Cainzos-Achirica’s comments on possible adverse effects ignore pharmacokinetic considerations.

## 10. Editorial on Vitamin C in JAMA by Kalil

A third recent editorial in JAMA [151] discussed studies on vitamin C and sepsis, and commented on the VITAMINS trial [152]. The editorial is again misleading in several ways [153].

Kalil wrote: “the very low levels [of vitamin C] characteristic of scurvy...are uncommon in sepsis” [151]. This comment suggests that Kalil was not familiar with the reports of experimental vitamin C deficiency studies. In their small vitamin C deprivation trial, Hodges et al. reported that ecchymoses started to appear when plasma vitamin C levels fell below 17 μM (0.3 mg/dL), and petechiae when vitamin C fell below 14 μM (0.24 mg/dL) [154]. Many other scurvy symptoms started to appear when vitamin C levels fell below 9 μM (0.16 mg/dL). Kalil does not describe what he specifically means with “the very low levels characteristic of scurvy”, and there are no references in his comment.

At the baseline of the CITRIS-ALI trial, one quarter of patients had vitamin C levels below 11 μM in the placebo group, and below 8 μM in the vitamin C group [115]. When one quarter of sepsis patients have vitamin C levels below the levels when many scurvy symptoms start to appear, and many more have vitamin C levels below 17 μM, when some initially healthy people start to get scurvy symptoms, it is misleading to claim that scurvy levels are particularly uncommon in sepsis patients. Very low vitamin C plasma levels, such as those found in scurvy patients, are not uncommon in hospitals [155,156,157,158,159,160,161,162].

Furthermore, when considering the diagnosis of scurvy, a focus on plasma vitamin C levels is simplistic. From their experiment on vitamin C deficiency, Hodges concluded that “by the time the [vitamin C] pool size has decreased to less than 300 mg and the daily rate of catabolism to less than 9 mg, signs and symptoms of frank scurvy become obvious. A distressing feature is the lack of precision of serum ascorbic acid levels. According to most authorities, deficiency appears after the serum level has fallen below 0.2 mg/100 mL (11 μM), yet several men in these studies had obvious scurvy at a time when their serum levels were above this value” [163].

Kalil wrote [151]: “The reported mortality rates were in favor of vitamin C in the other 2 trials [115,124], although in one of those trials [124], the sample size was small (28 patients)”. Logically, a small sample size cannot explain the statistically significant benefit (*p* = 0.01; based on 2/14 vs. 9/14) reported by Zabet [124]. A small sample size can lead to a false negative result because of low statistical power. However, the risk of a false positive finding is not increased when the sample size gets smaller.

Kalil continued the same sentence: “...and in the other trial [115], mortality was not the primary outcome and lack of adjustment for multiple testing weakened the inference for the mortality outcome”. See Section 6 for a discussion of the flaws in this reasoning.

Kalil further wrote: “Vitamin C has now been evaluated as a treatment for sepsis and septic shock, either alone or in combination, in 8 RCTs...Of the 8 RCTs, 6 (including a total of 633 patients) showed no significant effect of vitamin C on mortality [152,164,165,166,167,168]”.

Although Kalil first speculated that the small sample size might explain a positive result (see above), here Kalil ignores the fact that “no significant effect” can simply be explained by the fact that the sample size was too small. Based on the confidence intervals, all six trials to which Kalil refers are non-informative about a possible 20% reduction in mortality in the vitamin C groups (Table 6). A 20% decrease in mortality can be clinically relevant when the intervention is a safe and very inexpensive essential nutrient, such as vitamin C. None of the confidence intervals of the six trials refutes such a potential effect.

Furthermore, two of Kalil’s so called “vitamin C” studies were not vitamin C studies. Galley administered vitamin E and N-acetylcysteine together with vitamin C [167]. Schneider administered vitamin E, selenium, and many other substances together with vitamin C [168]. Significant harmful interactions between vitamin C and vitamin E were observed on mortality [169] and on the incidence of community-onset pneumonia [170] among middle-aged males of the large ATBC Study. Therefore, vitamin E should not be assumed inactive when administered together with vitamin C. Thus, Kalil did not focus on trials that investigated vitamin C alone.

In addition, the statement by Kalil concerning the “6 [RCTs] (including a total of 633 patients)” is false. The correct number of participants in the six trials was just 443 (Table 6). When focusing on the four trials that were actual vitamin C trials, there were just 355 participants. Thus, by referring to non-informative small trials with wide confidence intervals, including trials that examined vitamin combinations, and claiming more participants than actually had been investigated, Kalil makes it appear that the potential benefit of vitamin C for sepsis patients has been confidently refuted. 

A further way in which Kalil misleads readers is by focusing only on mortality. There are other outcomes that are relevant for critically ill patients. A meta-analysis of ICU trials calculated that “In 12 trials with 1766 patients, vitamin C reduced the length of ICU stay on average by 7.8% (95% CI: 4.2% to 11.2%; *p* = 0.00003)...In three trials in which patients needed mechanical ventilation for over 24 h, vitamin C shortened the duration of mechanical ventilation by 18.2% (95% CI 7.7% to 27%; *p* = 0.001)” [123]. Another meta-analysis calculated that “In the non-US cardiac surgery trials, vitamin C decreased the length of hospital stay by 12.6% (95% CI 8.4–16.8%) and ICU stay by 8.0% (95% CI 3.0–13.0%). The US trials found no effect on hospital stay and ICU stay” [122]. These findings were dismissed by Kalil [151]. Although they are not specifically about sepsis, they are relevant for all critically ill participants in general, including sepsis patients. Even if mortality is not decreased, shorter ICU or hospital stays are themselves clinically relevant outcomes.

Finally, Kalil wrote that “considering the available evidence from more than 2000 patients in both observational and randomized studies, there is insufficient equipoise to continue enrolling more patients in sepsis trials involving high-dose vitamin C administration” [151].

In the beginning of his editorial, Kalil is critical of observational studies because of possible baseline imbalances, yet here he uncritically pools observational studies and RCTs together. Given the concerns around bias in observational studies, they should be kept separate from the RCTs. The controlled trials on vitamin C alone, to which Kahil referred, included just 355 participants (Table 6), which is far short of the 2000 participants claimed in his editorial.

Finally, Kalil did not specify what he considered to be the minimal clinically relevant effect, and he did not carry out any sample size calculations for an appropriately powered vitamin C trial. Assuming a baseline risk of death of 20%, as in the VITAMINS trial [152], and considering a 20% decrease to be clinically relevant, we calculated the sample sizes needed to detect a difference between trial arms. The effect of a 20% decrease in risk corresponds to a 16% mortality rate in the vitamin C group. For a study with 80% power, there should be 1447 participants in each group [171]. Given the safety and low cost of vitamin C, a 10% decrease may also be a clinically meaningful effect, and that would lead to a sample size of 6039 per group [171]. Kalil does not postulate that a 10% or 20% benefit from vitamin C is too low to justify its usage. Obviously, 355 participants in the four vitamin C only trials listed by Kalil are not sufficient to test whether vitamin C has any clinically relevant effects.

## 11. Why Is There Bias against Vitamin C for Conditions Other Than Scurvy?

In the first half of the 20th century, a large number of papers were published in the medical literature on vitamin C and infections. Many physicians were enthusiastic about the beneficial effects of the vitamin, e.g., [172,173,174,175,176,177,178,179,180,181,182], but in the latter part of the 20th century, interest waned. One would expect that this could only be because large-scale controlled trials showed that vitamin C was ineffective. However, many rather large trials have demonstrated that vitamin C is effective [1,2,3,4,5,6,7,8,9,24,25,26,27,28,29,30,31,32] (Table 1). We postulate that there are several reasons why the interest in vitamin C and infections declined.

First, many medical researchers are not familiar with vitamin C biology. It was identified as the explanation for scurvy, which was considered a disease of the connective tissues. Evidently, it seemed illogical to consider that a substance that participates ‘only’ in collagen metabolism might also have an effect on infections. However, the biochemistry and actions of vitamin C are complex and not limited to collagen metabolism [183,184,185,186,187,188,189]. It also participates in the synthesis of noradrenalin, carnitine, nitric oxide, and in the terminal amidation of several neuropeptides. There are over 100 known neuropeptides, and over half of them require amidation to achieve full biological activity, such as vasopressin, oxytocin, gastrin, calcitonin, neuropeptide Y, and substance P. Vitamin C hydroxylates specific proline residues in hypoxia-inducible factor-I, which regulates hundreds of genes [185,186,187], and it also participates in the demethylation of DNA and histones, and thereby influences the epigenome [190,191,192]. It has been estimated that vitamin C may demethylate over 1000 genes in embryonic stem cells [190]. In addition, vitamin C is a major water-soluble antioxidant, and thereby can have a wide range of nonspecific effects [193]. Because of its complex and wide-ranging biochemistry, vitamin C also has various effects on the immune system [194,195,196].

Secondly, antibiotics were introduced in the mid-20th century. They have specific, and sometimes very dramatic, effects on bacterial infections, and therefore are a much more rational choice of first line drugs for patients with serious bacterial infections than vitamin C. Thus, it seems possible that the emergence of antibiotics overshadowed vitamin C when considering potential treatments for infections. Nevertheless, antibiotics are not effective against viral infections, and vitamin C and antibiotics are not incompatible.

Thirdly, three papers were published in 1975 which were particularly strong in leading to the loss of interest in vitamin C and the common cold in mainstream medicine (Table 2 and Table 3; Figure 2). It seems likely that they substantially increased the negative attitude towards vitamin C for other infections and medical conditions.

Fourthly, poor selection of participants is a particular issue in studies looking at the effectiveness of vitamins. Padayatty and Levine pointed out that “Many studies of vitamin supplements are flawed because vitamin concentrations at enrollment are usually not measured”. They illustrated the problem with an analogy: “studies to test antihypertensive therapy would not be done without measuring blood pressure at enrollment. To treat everyone regardless of blood pressure would be illogical. However, this strategy has been persistently pursued in evaluating vitamin supplements” [197].

A good example of this problem in the vitamin C and infection field is a cohort study of male health-care workers. Merchant et al. analyzed the relationship between dietary vitamin C intake and the incidence of pneumonia and found no correlation [198]. However, in that cohort of health-conscious people, the lowest decile of vitamin C intake was 95 mg/d and the overall median intake was 218 mg/d. Yet, the overall median vitamin C intake among the general US population is around 90–100 mg/d [199] (pp. 416–419, 432–435). Thus, the selection of participants in the Merchant study was severely biased when compared with the general population, and particularly with people of a lower socioeconomic status.

In the early literature, low vitamin C levels were associated with increased risk and severity of pneumonia [24,28,32,172,173,174,175,176,177,178,179]. For example, Glazebrook and Thomson estimated that the schoolchildren in their trial received only 10–15 mg/d of vitamin C from food, and vitamin C supplementation significantly decreased the incidence of pneumonia in that population [29,178]. A meta-analysis of males with particularly low dietary vitamin C intake (around 50 mg/d) found that vitamin C supplementation decreased the incidence of common cold episodes by 30% (95% CI 19–40%) [8,9]. Thereby, it would seem relevant to compare low vitamin C intakes (e.g., 10–50 mg/d) with average or high intakes (e.g., 100–200 mg/d), with the hypothesis that particularly low intakes of vitamin C might increase the risks of contracting pneumonia or the common cold.

Vitamin C intakes lower than 50 mg/d are not particularly rare. According to the NHANES III survey in the USA, 5% of males aged 51–70 years had a vitamin C intake of less than 47 mg/d [199] (p. 416). In the CSFII survey in the USA, 5% of males aged 51–70 years had a vitamin C intake of less than 28 mg/d [199] (p. 432), and in Canada, 5% of males aged 51–70 years had vitamin C intakes of less than 23 mg/d [199] (p. 438). Even lower levels are common in less developed countries, which also have higher rates of pneumonia [28,200]. Thus, comparisons within the high intake range from 95 mg/d upwards, such as the Merchant study, are inconclusive about the possible effects of supplementation for people with low vitamin C intakes.

Unfortunately, for the average reader, Merchant’s study may give the impression that the findings about vitamin C and pneumonia are important, as there were 38,378 participants with 145,878 person-years of follow-up [198]. However, the large number of participants does not compensate for the fact that the group with the lowest vitamin C intake in that study actually had a high vitamin C intake.

This problem is also seen in the common cold trials. Karlowski et al. [33] studied NIH employees. It seems evident that they were much more compliant in following trial instructions than less health-literate people. However, it seems also evident that they had vitamin C intakes well above the average, and therefore the estimate of benefit might be biased downwards when considering the general population [47]. As to the possible importance of baseline vitamin C intake level, the Anderson (1972) [53] trial is important, as it found that vitamin C supplementation reduced the ‘total days indoors’ by 48% among participants who consumed <3 oz (0.9 dL) of fruit juice (a common dietary source of vitamin C), whereas the reduction was only 22% among those who consumed more juice. A similar modifying effect with fruit juice was seen in the therapeutic trial by Anderson (1975) [201].

Finally, a particularly large RCT, with 14,641 participants followed for eight years, examined the effect of vitamin C on the incidence of cardiovascular diseases and total mortality and found no benefit [202]. However, the participants were male US physicians. There was no description of baseline vitamin C plasma levels or dietary intakes, but it seems likely that they were comparable to those reported by Merchant [198]. Vitamin C supplementation may or may not be beneficial, e.g., for geriatric inpatients with documented low vitamin C levels [203], but a study with physicians with a mean age of 64 years does not test such a hypothesis.

## 12. Socio-Political Origins for the Bias against Vitamins

In addition to the more specific explanations discussed above, there are also socio-political roots to the bias against vitamin C supplements. Rima Apple reviewed the history of vitamins in the USA in the 20th century and described a number of interesting patterns in the discussions about vitamins [204,205,206,207,208]. She did not take a particular stand on the question of whether vitamins are useful, harmful, or neutral, but instead analyzed the political views and discussions on the topic.

According to Apple, the concern about America’s nutrition was not a concept generated by pharmaceutical companies pushing vitamin supplements. By the late 1930s, several studies documented the nation’s poor diet, including eating vitamin poor foods, modern processing destroying parts of vitamins, and poor diets due to the Great Depression. These issues were well reported in the popular press in the early part of the previous century, and this context led to the origin of the vitamin industry. Thus, while many of the more recent advertisements are exaggerated and mislead consumers, the concern about nutrition among lay people originated from scientific findings that were reported in the popular press. Apple also reported that many researchers found that workers receiving vitamins had considerably lower rates of absenteeism, stayed in their jobs longer, and even scored higher on their merit ratings [204] (pp. 3–10).

Apple points out that Linus Pauling, a chemist, biochemist, and peace activist, was not the first to consider vitamins for preventing and curing the common cold. Popular literature and advertisements linked subclinical vitamin deficiency and respiratory infections much earlier, and that was justified by papers in medical literature, e.g., [172,173,174,175,176,177,178,179,180,181,182]. In any case, Pauling received two Nobel Prizes and he was widely featured in the popular media. Spokespersons for the medical profession and nutrition researchers resented Pauling’s involvement. They believed that megadoses of vitamin C, at minimum, were ineffective and a waste of money, and at worst dangerous, and the popularity of a theory proposed by a non-physician weakened their position of authority in health matters. The altercations between Pauling and his opponents brought the controversial nature of the science of vitamins directly to the American public [204] (pp. 74–84).

While the public embraced Pauling’s claims, the medical profession rejected both his theory and his expertise. Even before his book *Vitamin C and the Common Cold* [3] was officially released, physicians began to assail his authority, criticizing both his scientific explanations and his right to make them. Physicians who opposed him—and they were many and vocal—attacked not only his rationale but also his person. Their resort to ad hominem invective weakened their own claims to be objective, dispassionate observers of medical research, and undoubtedly attracted even more attention to Pauling and his cause.

As one example, in a 1975 issue of JAMA, Philip L White from the AMA Department of Foods and Nutrition wrote that “the arguments in support of Senator Proxmire’s bills have been provided by those who stand to gain the most financially from the unrestricted sale of vitamins. Dr. Pauling has been a spokesman for one of those groups” [209]. Pauling wrote a letter to the editor to correct that “this statement is not true. I have not been a spokesman for any group standing to gain financially from the unrestricted sale of vitamins. The statement that I have been a spokesman for one of those groups is damaging to me, and I ask that this letter be published in order to rectify this damage as far as possible” [210].

According to Apple, these ad hominem attacks on Pauling made his opponents look petty. Their position was further undermined by the reams of conflicting data published in the popular press. If physicians, those who put themselves forth as experts, cannot agree, then maybe Pauling, a brilliant scientist, had a point [204] (p. 84).

In the early part of the last century, vitamin proponents and opponents both used scientific justifications for their views, though selecting different studies to illustrate their arguments. However, in the 1970s, there was a large change in culture and proponents had a further strong argument, namely, the consumer’s right to choose vitamin products without government interference [204].

Over several decades in the early 20th century, the FDA tried to tighten restrictions for selling vitamins, but for various reasons the proposals were not a great success. In the 1970s, the FDA tried again to regulate the sale of vitamins, but this led to tens of thousands of letters to the FDA and to Congressional representatives. According to Apple, FDA officials did not recognize the significance of this correspondence. However, the sheer volume of the public response should have alerted them to the power of the vitamin-taking consumer [204].

The consumers, who were also voters, criticized the FDA for ‘Big Brotherism’. Over and over, letter writers accused the agency of ‘treating the American people like children’. According to Apple, the actions of the FDA and much of its self-defensive publicity suggested that its officials believed the American consumer was incompetent, easily and unthinkingly swayed by advertising hype. Though the agency, secure in its own beliefs, gave vitamin-taking consumers little satisfaction, Congress was more willing to listen to the public’s concerns about ‘health freedom.’ By 1976, the Proxmire Amendment denied the FDA the role the agency had claimed. With reasons based on consumer’s rights and contemporary science, lawmakers prohibited the FDA from regulating vitamin products, including the potency, number, combination, amount, or variety of vitamins in products, unless the agency could prove that the product was ‘intrinsically injurious to health’ [204] (pp. 144–178).

## 13. Bias against Micronutrients in Academic Medicine

Another informative analysis of the attitudes of mainstream medicine towards vitamins was by Goodwin and Tangum [211]. They also did not take a stand on the question of whether vitamins were useful or not, but analyzed the biases in mainstream medicine, noting that it is not proof of bias to be concerned about toxicity or to be skeptical of claims of efficacy. Bias occurs when concern and skepticism are applied selectively to different but comparable topics.

They concluded that “Our thesis is that throughout much of the 20th century, American academic medicine was resistant to the concept that micronutrient supplementation might prove beneficial...This resistance is evident in several ways: (1) by uncritical acceptance of bad news about micronutrient supplements; reports of toxic effects were rarely questioned and widely quoted; (2) by the scornful, dismissive tone of the discussions about micronutrient supplementation in textbooks of medicine, a tone avoided in most medical controversies; and (3) by the skeptical reaction greeting any claim of efficacy of a micronutrient, relative to other therapies; indeed, most claims were simply ignored”. Several examples were described to support those conclusions [211] (p. 2187).

In their analysis of bias against vitamins, Goodwin and Tangum reviewed assertions over time in the two major textbooks of medicine in the USA: *Cecil’s Textbook of Medicine* and *Harrison’s Principles of Internal Medicine*. Both had been published in 12 different editions between 1950 and 1992. Goodwin and Tangum reasoned that the textbooks could be presumed to represent established opinions and how those opinions changed over time.

In Harrison’s text, routine use of multiple vitamins was condemned in the 1950s, 1960s, and 1970s. Cecil’s text evolved over time. The editions published prior to 1960 were positive towards vitamin use, but by 1963, the positive comments had vanished, and the treatment of multiple vitamin supplements was similar to that of Harrison.

Goodwin and Tangum reported that, over the preceding decades, there had been many areas of medical practice about which uncertainty and controversy existed, all well covered in the various editions of these two major textbooks, but in none of these discussions did one encounter the contemptuous descriptions found in the discussions of multiple vitamins.

Goodwin and Tangum also pointed out that there are many factors that influence the adoption of new medical treatments, including efficacy, toxic effects, and cost, but they also referred to financial incentives due to patent protection, which usually stimulate the aggressive marketing of new pharmaceuticals. These financial incentives were lacking in the case of micronutrients. However, these factors do not explain the anger and scorn illustrated in the quotations from medical textbooks given in their paper. Goodwin and Tangum rightly asked: “Where did the emotion come from? Why did academic medicine deploy the strongly negative language of denunciation against proponents of vitamin supplements?” [211].

## 14. The Great Influence of AMA and JAMA

Most of the papers discussed in this review are from JAMA. This should not be interpreted to mean that JAMA is the only forum that has been biased against vitamins. However, JAMA is the journal for the AMA, which serves as a thermometer for the beliefs of opinion leaders in mainstream US and global medicine. JAMA also has very wide circulation and significant prestige, and itself influences the opinions of large numbers of physicians. For example, the CITRIS-ALI paper in JAMA [115] has been viewed 139,000 times and cited 295 times, and the COVID A to Z trial [133] has been viewed 428,000 times and cited 29 times so far (11 December 2021). Publishing those studies misleadingly as negative trials with associated negative editorials is in line with Goodwin and Tangum’s view that “negative results [on micronutrients] are published in the best journals, with a celebratory tone to the accompanying editorials” [211] (p. 2190).

It is also perplexing that none of the authors of the three editorials that we discuss in Section 8, Section 9 and Section 10 has conducted any of their own scientific work on vitamin C, as far as we can determine from PubMed searches. Surely, if a journal is serious about evidence, editorials should be invited from researchers who have significant scientific experience in the field.

One high level example of the biases of the AMA against vitamin C is the formal statement of the Council of Scientific Affairs of AMA that “There is no reliable evidence that large doses of ascorbic acid prevent colds or shorten their duration”, which was published in JAMA in 1987 [83] (p. 1934) and was entirely based on the Chalmers (1975) review [82] (Table 3) (see Section 3). To our knowledge, this statement was not withdrawn after the Chalmers review was shown to be flawed eight years later [86]. Thereby, the AMA and JAMA have been misleading readers for over three decades so far. It is particularly concerning that none of the researchers at the Council of Scientific Affairs of the AMA had looked at the vitamin C and common cold field in any detail to assess the validity of the Chalmers (1975) review. The errors in that review are so significant that even a superficial look at the literature would have shown that the review is not accurate, see e.g., Table 4. One would expect that a council that forms strong statements intended for very wide readership would first do its homework before publishing such statements.

In addition to the biases on vitamin C and respiratory infections that we have described in our paper, the publication policy of JAMA was also unusual in relation to the claimed breakdown of vitamin B_12_ by vitamin C. In 1974, JAMA published a study by Herbert and Jacobs that claimed that vitamin C causes the breakdown of vitamin B_12_ [212]. Pauling described the subsequent chain of events as follows: “When Newmark and his coworkers found that the claim could not be substantiated, and that in fact vitamin C does not destroy the vitamin B_12_ in the food, they sent their paper to the editor of JAMA, which seems clearly to be the place where the correction should be published. He held it for half a year, and then refused to publish it, thus delaying its publication in another journal and preventing many of the readers of the original article by Herbert and Jacobs from learning that their results were incorrect. These actions suggest that the AMA works to protect American physicians from information that runs counter to its own prejudices. The evidence indicates that the AMA is prejudiced against vitamin C” [109,110]. In 1976, Newmark’s study was published in a smaller journal, which does not reach the wide readership of JAMA [213]. Five years after the publication of the Herbert and Jacobs’ vitamin B_12_ paper, JAMA finally published a short paper by Newmark discussing the flaws in Herbert’s study [214]. Thus, for five years the readers of JAMA were misled that vitamin C causes breakdown of vitamin B_12_. There is no evidence of this, and “results of in vivo studies in human subjects have shown that vitamin C intakes up to 4 g/day did not induce vitamin B_12_ deficiency” [199] (p. 158).

As described in Section 5, the two influential papers on vitamin C and the common cold were published in the 10 March 1975 issue of JAMA [33,101]. Five months later, in the 11 August 1975 issue, JAMA published two other negative papers on vitamins [215,216]. Evidently, these two papers further strengthened the rejection of interest in vitamin C (Figure 2). In a letter-to-the-editor, Sackler pointed out the bias in the two papers as follows: “I was distressed to note the ad hominem attack on Linus Pauling by Thomas H. Jukes (233:550, 1975) that appeared with an editorial (233:538, 1975). It does not serve the scientific method to practice the discredited procedure of guilt by association…The article and editorial do not serve the cause of scientific debate when they omit any reference to a significant and growing number of published papers or when they totally omit reference to Pauling’s analysis of data in those reports on ascorbic acid and the common cold” [217]. Thus, the bias in the editorials in JAMA on vitamin C, which we describe in Section 8, Section 9 and Section 10, is not a new problem.

The negative view of the AMA against vitamins traces back a century. In her history of vitamins in the USA, Apple described that “the American Medical Association (AMA) was most critical of the emerging [vitamin] industry. In articles and in editorials, starting as early as 1922, the organization called the commercial hype surrounding vitamins ‘a gigantic fraud’” [204] (p. 8). Over and over again, the AMA campaigned against vitamin supplements, insisting that a diet lacking vitamins would give rise to gross vitamin-deficiency diseases, and that these conditions were the concern of the doctor. According to AMA articles, vitamin pills were within the purview of the physician, not the layperson. In Apple’s view, clearly, commercial vitamin pills undercut the authority of the medical practitioners to direct the patient’s health. Thus, concerns about power structure in respect to the new substances and who is in charge of the health of the population may have been one of the causes for the bias against vitamins.

Furthermore, according to Apple, leaders of the AMA frequently worked with representatives of the FDA to denounce vitamin therapy as ‘fraud’. They cautioned that little evidence existed to document the power of vitamins, except in clear cases of vitamin deficiency. Physicians dismissed the idea that vitamin supplementation could benefit the cold sufferer. In 1961, a popular health magazine produced by the AMA simply wrote off any connection as ‘pure superstition’ [204] (p. 77).

In a history of efforts to regulate dietary supplements in the USA [218] (pp. 272–273), Swann summarized the history of the AMA towards vitamins as follows: “By the early 1920s the American Medical Association (AMA) began voicing concern with the proliferation of vitamins. This was not much of a stretch for the AMA, folding vitamins into the legion of patent medicines against which they had railed for decades—both types of products having removed or at least decreased the self medicator’s contact with a physician; not that there was a level of scientific uncertainty attendant to many of the claims made for them. The AMA’s journal carried a report from a University of Illinois researcher whose analysis of vitamin studies led him to the conclusion that, in the absence of a vitamin deficiency, the ‘benefit from an excess of any [vitamins] seems most improbable, and we lack proof that such is the case’. The association criticized the ‘irresponsible’ and ‘distorted’ advertising for vitamins in popular magazines, and reminded their readership of ‘the recent form of quackery that has used the remarkable story of the vitamins in physiology as a device to promote the sale of nostrums’. It was the beginning of a battle that would last the rest of the century”.

Finally, based on their historical analyses of rejections of highly efficacious therapies, Goodwin and Goodwin concluded that one important reason for rejection was inconsistency with the then current biological theories [219,220]. They also concluded that “if a treatment bypasses the medical establishment and is sold directly to the public...the temptation in the medical community is to accept uncritically the first bad news that comes along” [220]. This seems to be a contributing factor to the lack of interest in vitamin C in mainstream medicine.

## 15. Biases on Vitamin C and Cancer

Our paper is primarily focused on vitamin C for infections, but we briefly comment on the controversy about vitamin C and cancer for two reasons. Firstly, after his work on vitamin C and the common cold [1,2,3], Pauling became interested in the effects of vitamin C on cancer. Secondly, there is a comprehensive sociological analysis of the severe biases in the assessment of vitamin C’s effects on cancer [221,222,223,224,225,226].

In the 1950s, the Canadian physician William McCormick recognized that certain changes in tissues in scurvy are similar to changes observed in cancer tissues. He also pointed out that cancer patients were usually depleted of vitamin C, and proposed that vitamin C may play a role in cancer [227]. In the early 1970s, Scottish surgeon Ewan Cameron became interested in the idea that vitamin C may have an effect on cancer, and began a collaboration with Linus Pauling. They published two studies at the Vale of Leven hospital and reported that cancer patients who were administered vitamin C lived longer than historical controls not given the vitamin [140,141]. Cameron and Pauling had applied for funding from the NIH for randomized trials, but all their applications were rejected, and therefore they were unable to undertake trials with better methodology [142]. Although there can be biases in comparisons with historical controls, many alternative explanations for the published results seemed less plausible than vitamin C having an effect [228]. Cameron and Pauling also wrote a lengthy review of the biological rationale explaining how vitamin C might affect cancer [143].

The topic subsequently became popular in the USA in the 1970s, and two randomized trials were carried out. The first trial was carried out by Creagan et al. [229]. No benefit was seen from vitamin C in that trial.

In a letter-to-the-editor, Pauling wrote that “the paper by Creagan et al. misrepresents the Vale of Leven study in stating that 50 of the 100 ascorbate-treated patients, rather than only four, had received prior chemotherapy” [144]. Pauling continued his critique as follows: “Answering a letter from me, Dr. Moertel wrote on 6 May 1977, ‘I feel it is essential in our first evaluation of ascorbic acid that we make every effort to duplicate the conditions which existed in the clinical trial conducted by Cameron but to use a randomized prospective study design rather than the historical controls employed by Cameron,’ and on August 8, 1978, I sent to Dr. Moertel a letter containing the following sentences: ‘In my last letter to you I pointed out to you that the patients studied by Dr. Cameron had not received chemotherapy. The cytotoxic drugs damage the body’s protective mechanisms, and vitamin C probably functions largely by potentiating these mechanisms. Accordingly, if you hope, as you stated in your letter, to repeat the work of Cameron as closely as possible, you should be careful to use only patients who have not received chemotherapy…Otherwise the trial cannot be described as repeating the work of Cameron’ ” [144].

The second randomized trial was carried out by Moertel et al. [230]. In its introduction, the report of the second trial described that “the present study was undertaken to test the thesis put forth by Cameron and Pauling that high-dose vitamin C is effective therapy for advanced cancer patients who have had no previous exposure to chemotherapy”. The second trial also found no benefit from vitamin C.

However, Pauling pointed out serious limitations with the Moertel trial. For example, the Moertel “study provides no information about the value of a continued intake of 10 g per day of vitamin C in extending survival time, because none of the patients died while taking the vitamin” [145]. The median duration of vitamin C administration was just 2.5 months, whereas the median duration of survival was about 9 months [230]. Administering vitamin C for such a short period seems to be poor trial design. Pauling had serious concerns about this trial based on the fact that it failed all three biostatistical criteria formulated for the validity of clinical trials of cancer treatments [145]. In a citation search, we found no indication that Pauling’s statistical criticism [145] was challenged or refuted in later literature.

Based on the randomized trials by Creagan [229] and Moertel [230], vitamin C was refuted as a cancer treatment in mainstream medicine. However, the study design (RCT vs. historical controls) is not the only important difference between the Creagan and Moertel trials and the Cameron and Pauling studies. There are also important biological differences between the trials.

Moertel measured the urinary vitamin C excretion of six placebo participants and reported that “five had negligible levels that were within the range of normal controls for our assay method (≤0.55 g per 24 h)”, while one of the six placebo patients had vitamin C excretion over 0.55 g/d [230]. This is quite puzzling. The recommended dietary intake level of vitamin C in 1985 was 0.06 g/d [65] (p. 75). Therefore, a meaningful trial about additional vitamin C should administer about 0.06 g/d of vitamin C to the control group if the goal of the trial is to examine whether a dose higher than the recommended dose is better. Urinary vitamin C excretion is essentially nil when intake is 0.06 g/d, whereas excretion of 0.55 g/d indicates that the dietary intake must have been over ten times the recommended intake [231,232]. Moertel did not report the distribution of the urinary vitamin C levels, so it is not known how many had excretion close to 0.5 g/d. There was also no explanation of whether the diet was particularly rich in vitamin C, or whether the placebo patients may have been taking vitamin C outside of the trial protocol.

Another important biological difference is the route of vitamin C administration to vitamin C participants. In the Moertel trial, 10 g/d of vitamin C was administered orally [230], whereas in the Cameron studies, 10 g/d was administered intravenously for a week [140]. In healthy subjects, oral administration of 10 g/d of vitamin C leads to a plasma level of about 200 μM, whereas the same dose intravenously leads to a peak plasma level of about 5000 μM [149,232]. Thus, high plasma levels in the placebo group and low plasma levels in the vitamin C group could have led to a false negative result in the Moertel trial for biological reasons. The fact that the study was an RCT did not guarantee the biological relevance of the trial. Consequently, the Moertel trial was also inconclusive about the potential benefits of intravenous vitamin C for cancer patients. This has similarities to the study by Merchant, in that the vitamin C levels being compared are not meaningful (Section 11).

Over the decades since the 1980s, the biological rationale for vitamin C for cancer has not diminished; rather, an increase in biochemical understanding has made clinical effects even more plausible [196,232,233,234,235,236,237,238,239]. A few studies have been published indicating that vitamin C might be beneficial, at least for some cancer patients. Following the National Cancer Institute Best Case Series guidelines, Padayatty et al. reported three cases of advanced cancers who had unexpectedly long survival times after receiving 15–65 g/d of vitamin C intravenously twice per week [240]. Hoffer et al. also described three patients with different types of cancer who experienced unexpected transient stable disease, increased energy, and functional improvement after intravenous vitamin C [241]. In an RCT, Ma et al. found that adverse events caused by the chemotherapeutic agents carboplatin and paclitaxel for ovarian cancer patients were significantly reduced with intravenous vitamin C administration, with a target peak concentration of 20,000 to 23,000 μM [242].

Further research will show whether vitamin C has beneficial treatment effects on cancer or not. In any case, the discussion around vitamin C for cancer is very similar to that around vitamin C for the common cold, in which case one RCT [33] also caused widespread dismissal of the possible benefits of the vitamin and discouraged further research (Figure 2; Table 2). Five decades after the Cameron and Pauling papers [140,141], we still do not know if intravenous high-dose vitamin C is beneficial for a proportion of cancer patients.

Nevertheless, the history of the vitamin C and cancer field is fascinating from the point of view of documented biases in the attitudes towards the vitamin. Evelleen Richards published a thorough and thoughtful sociological analysis about vitamin C and cancer, in which she compared the attitudes and arguments of physicians to three putative cancer medicines: 5-fluorouracil, interferon, and vitamin C [221,222,223,224,225,226]. She documented unambiguous bias against vitamin C when compared with the conventional treatments, neither of which turned out to be efficacious at the end of the research [221,222]. Given the poor-quality evidence for many conventional cancer drugs that are allowed to enter the market [243,244,245,246], the strong negative attitude in mainstream medicine against vitamin C appears particularly concerning. Where do such strong emotions come from that have prevented appropriate investigation of vitamin C for cancer?

## 16. Conclusions

The belief that vitamin C is ‘ineffective’ against the common cold is widespread. For example, a survey of general practitioners in the Netherlands found that 47% of respondents considered that homeopathy is effective against the common cold, but just 20% of the respondents considered that vitamin C was effective [247]. Given the wide range of demonstrated biochemical effects of vitamin C, many of which have been known for more than half a century, and the benefits observed in numerous placebo-controlled trials in the 1970s, it is concerning that so few general practitioners believed vitamin C to be effective; yet, that is what the survey found. This is one illustration of how deeply physicians have been influenced by the flawed presentations of the vitamin C field in textbooks and major journals (Table 2 and Table 3).

There has also been strong negative language around vitamin C, which does not seem to be present to the same extent elsewhere. For example, there is no rationale for prescribing antibiotics for viral infections, yet this regularly occurs. While the practice is discouraged, it does not attract the same strong emotion as vitamin C use, even though there is considerable evidence for the benefits of vitamin C, whereas there is no evidence for antibiotic use for viral infections.

A further puzzling issue about vitamin C is its labeling in mainstream medicine. Under the classifications used by the NIH and the Cochrane Collaboration, the usage of vitamin C for preventing and treating diseases other than scurvy falls under the category of complementary and alternative medicine [248,249,250]. However, this categorization does not reflect the level of evidence for vitamin C, but instead reflects its low level of acceptance in the medical community. Unfortunately, such a classification may further amplify the inertia and prejudices against the vitamin [251].

In this review, we have demonstrated significant bias in many influential papers on vitamin C. We have shown that many influential papers have been uncritically cited in textbooks and reviews, assuming that they are scientifically valid, when in fact many of them have serious flaws. Influential authors have referred to the papers without undertaking any critical appraisal themselves. We have proposed conceptual explanations for the long-lasting and deep bias against vitamin C. This bias is unfortunate because vitamin C is safe and inexpensive, and therefore even reasonably small treatment effects are well worth taking into consideration.

## Figures and Tables

**Table 4:** Errors in Chalmers’ presentation of the Karlowski (1975) trial results.

	Ascorbic Acid		Placebo		
	No. of Subjects	Mean Duration (Days)	No. of Subjects	Mean Duration (Days)	Difference in Duration (Days)
**Chalmers (1975) review [82]:**					
Karlowski (1974)	101	6.80	89	6.30	+0.50
**Correct data [33,34]:**					
Karlowski (1975)	57	5.92	46	7.14	−1.22 (*p* = 0.046)

In the Chalmers (1975) review, half of the Karlowski (1975) trial “placebo group” participants were actually vitamin C participants (43 of the 89). The common cold duration in Chalmers’ “placebo” group was actually the mean of two vitamin C groups [86]. Correct data shows the findings for the 3 + 3 g/day and 0 + 0 g/d (placebo) groups. All four groups are shown in Figure 1, and analyzed in [47].

**Table 5:** Coulehan (1974) report of the number of children who were never ill during the study.

	Vitamin C	Placebo
Total No. of children	321	320
No. of children ‘never ill on active surveillance’	143	93
Comparison of groups:		
*p* (Fisher test)	0.000058	
*p* (χ^2^ test)	0.000048	
RR (95% CI)	1.53 (1.24–1.89)	

The rate ratio (RR) indicates that 53% more of the children were ‘never ill on active surveillance’ in the vitamin C group. Data are from table 4 of the Coulehan (1974) report [107]; see also [24] (p. 44). See Listing S3 for the calculations in the Supplementary Materials.

**Table 6:** Trials that indicated a lack of effect of vitamin C on sepsis according to Kalil.

Trial	Mortality in Vitamin C Group, 95% CI of the RR	No. of Participants			Notes
		Vitamin C	Control	Total	
Fujii (2020) [152]	0.69–2.0	107	104	211	
Ferron-Celma (2009) [164]	0.60–3.7	10	10	20	
Fowler (2014) [165]	0.32–1.5	16	8	24	
Nabil Habib (2017) [166]	0.36–1.2	50	50	100	Not negative ^(1)^
Galley (1997) [167] ^(2)^	0.69–2.1	16	14	30	400 mg vit E with vit C
Schneider (2011) [168] ^(2)^	0.37–2.7	29	29	58	Vit E and other substances with vit C
Total participants		228	215	443	
Total participants in actual vitamin C trials ^(2)^		183	172	355	

^(1)^ Nabil Habib reported: “There was a statistically significant difference in ICU stay between the two groups (*p* = 0.04)” [166], and thus it is not an unambiguously negative trial, but Kalil [151] does not mention that finding. ^(2)^ The Galley (1997) and Schneider (2011) trials administered other substances such as vitamin E together with vitamin C. Therefore, they do not measure the specific effect of vitamin C alone. In some contexts, vitamin C and vitamin E have had harmful interactions on clinical outcomes [169,170]. See Listing S6 for the calculation of the 95% CIs in the Supplementary Materials.

## Data Availability

Data for Figure 1 are available at [33,34], for Figure 3 at [115,116], and for Figure 4 at [133,134].

## Supplementary Materials

The following are available online at www.mdpi.com/article/10.3390/life12010062/s1, Listing S1: Selected list of papers that cite the Karlowski (1975) trial, Listing S2: Statistical calculations for Figure 1, Listing S3: Statistical calculations for Table 5, Listing S4: Statistical calculations for Figure 3, Listing S5: Statistical calculations for Figure 4, Listing S6: Statistical calculations for Table 6.
