# Supplementary material for: Bias against Vitamin C in Mainstream Medicine: Examples from Trials of Vitamin C for Infections
Source: Life (Basel). 2022 Jan 3;12(1):62. doi: 10.3390/life12010062 (PMC8779885; doi:10.3390/life12010062)
Supplement: Supplementary file 1 [file life-12-00062-s001.zip › life-1507004-supplementary.pdf]

Bias against vitamin C in mainstream medicine: examples from trials of vitamin C for infections

Harri Hemilä and Elizabeth Chalker

Supplementary file
https://www.mdpi.com/journal/life

Harri Hemilä, MD, PhD
Department of Public Health,
University of Helsinki, POB 41,
Helsinki, FI-00014, FINLAND.
E-mail: harri.hemila@helsinki.fi
https://www.mv.helsinki.fi/home/hemila

ver 2021-12-17

Contents **Page**

Listing S1: Selected list of papers that cite the Karlowski (1975) trial	2
Listing S2: Statistical calculations for Figure 1	26
Listing S3: Statistical calculations for Table 5	28
Listing S4: Statistical calculations for Figure 3	29
Listing S5: Statistical calculations for Figure 4	30
Listing S6: Statistical calculations for Table 6	31

Listing S1: Selected list of papers that cite the Karlowski (1975) trial

Karlowski, T.R.; Chalmers, T.C.; Frenkel, L.D.; Kapikian, A.Z.; Lewis, T.L.; Lynch, J.M.
Ascorbic acid for the common cold. A prophylactic and therapeutic trial.
JAMA. 1975, 231, 1038-1042.
https://doi.org/10.1001/jama.1975.03240220018013
https://pubmed.ncbi.nlm.nih.gov/163386
https://scholar.google.fi/scholar?cites=2736862160336687087

In total, our Web of Science search (2021 Nov) identified 185 citations to the Karlowski trial since its publication in 1975. Google Scholar lists 331 citations.

This list of 67 citations below is a selection of more important citations to the Karlowski trial. We show the contexts in which the trial has been cited. In most of the selected cases, the Karlowski trial has been cited as an evidence of the placebo effect in action. In some cases, the trial has been cited as evidence that vitamin C has no effects on the common cold. There are some cases when the author knew that the Karlowski trial was analyzed erroneous, yet the author still cited it as evidence of the placebo effect.

Title: **Measuring the success of blinding in placebo-controlled trials: Should we be so quick to dismiss it?**
Author(s): Webster, RK (Webster, Rebecca K.); Bishop, F (Bishop, Felicity); Collins, GS (Collins, Gary S.); Evers, AWM (Evers, Andrea W. M.); Hoffmann, T (Hoffmann, Tammy); Knottnerus, JA (Knottnerus, J. Andre); Lamb, SE (Lamb, Sarah E.); Macdonald, H (Macdonald, Helen); Madigan, C (Madigan, Claire); Napadow, V (Napadow, Vitaly); Price, A (Price, Amy); Rees, JL (Rees, Jonathan L.); Howick, J (Howick, Jeremy)
Source: JOURNAL OF CLINICAL EPIDEMIOLOGY Volume: 135 Pages: 176-181
DOI: 10.1016/j.jclinepi.2021.02.022
https://doi.org/10.1016/j.jclinepi.2021.02.022

“Aside from the importance of blinding itself, the importance of measuring (see Box 1) and reporting blinding success is apparent in various trials. For example, Karlowski, et al [29] compared Vitamin C with placebo for treating the common cold, and found Vitamin C to be apparently effective. However, because of the sour taste of Vitamin C and sweet taste of the lactose placebo pills, the trial was not successfully blinded. When the authors carried out a subgroup analysis in which they divided participants into those who remained blinded and to those who were not, they found that there was no benefit of Vitamin C in the blinded group. Although ideally the authors should have ensured both placebo and active intervention were adequately matched, this example still shows the importance of measuring and reporting blinding success. Otherwise, it would have been mistakenly concluded that Vitamin C was superior.”

Title: **The Long History of Vitamin C: From Prevention of the Common Cold to Potential Aid in the Treatment of COVID-19**
Author(s): Cerullo, G (Cerullo, Giuseppe); Negro, M (Negro, Massimo); Parimbelli, M (Parimbelli, Mauro); Pecoraro, M (Pecoraro, Michela); Perna, S (Perna, Simone); Liguori, G (Liguori, Giorgio); Rondanelli, M (Rondanelli, Mariangela); Cena, H (Cena, Hellas); D'Antona, G (D'Antona, Giuseppe)
Source: FRONTIERS IN IMMUNOLOGY Volume: 11 Article Number: 574029
DOI: 10.3389/fimmu.2020.574029 Published: OCT 28 2020
PubMed ID: 33193359
https://doi.org/10.3389/fimmu.2020.574029

> "However, other clinical studies with similar aims failed to demonstrate its efficacy (118–121)"
> [119=Karlowski]
> [120=Chalmers 1975; 121=Dykes-Meier 1975]
>
> **See comments by Hemilä in:**
> https://doi.org/10.3389/fimmu.2021.659001

Title: **Efficacy of vitamin C for the prevention and treatment of upper respiratory tract infection. A meta-analysis in children**
Author(s): Vorilhon, P (Vorilhon, Philippe); Arpajou, B (Arpajou, Bastien); Roussel, HV (Roussel, Helene Vaillant); Merlin, E (Merlin, Etienne); Pereira, B (Pereira, Bruno); Caballiot, A (Caballiot, Aurelie)
Source: EUROPEAN JOURNAL OF CLINICAL PHARMACOLOGY Volume: 75 Issue: 3 Pages: 303-311
DOI: 10.1007/s00228-018-2601-7 Published: MAR 2019
PubMed ID: 30465062
https://doi.org/10.1007/s00228-018-2601-7

> "However, later trials failed to corroborate this preventive effect of vitamin C supplementation [8–10]."
> [10=Karlowski]
> [8=Dykes-Meier 1975; 9=Chalmers 1975]
>
> **See comments by Hemilä in:**
> https://doi.org/10.1007/s00228-019-02733-x
> https://helda.helsinki.fi/handle/10138/318103
> https://doi.org/10.13140/RG.2.2.18773.14564
> https://helda.helsinki.fi/handle/10138/333365
>
> **The Vorilhon meta-analysis was retracted because of numerous errors:**
> https://doi.org/10.1007/s00228-021-03150-9

Title: **Vitamins C and D**
Author(s): Shader, RI (Shader, Richard I.)
Source: CLINICAL THERAPEUTICS Volume: 39 Issue: 5 Pages: 873-877
DOI: 10.1016/j.clinthera.2017.04.001 Published: MAY 2017
PubMed ID: 28420486
https://doi.org/10.1016/j.clinthera.2017.04.001
http://www.ncbi.nlm.nih.gov/pmc/articles/pmc7119064/

> "In 1996, Hemilä, a strong supporter of therapeutic roles for vitamin C, challenged the conclusions by several National Institutes of Health authors [10,11] that a placebo effect colored the interpretation of their findings"
> [10=Karlowski]
> [11=Chalmers 1975]
>
> **See comment by Hemilä in:**
> https://doi.org/10.1016/j.clinthera.2017.08.005
> https://helda.helsinki.fi/handle/10138/228957

Title: **Clinical Trials**
Author(s): Califf, RM (Califf, Robert M.)
Edited by: Robertson D; Williams GH
Source: CLINICAL AND TRANSLATIONAL SCIENCE: PRINCIPLES OF HUMAN RESEARCH, 2ND EDITION
Pages: 25-52
DOI: 10.1016/B978-0-12-802101-9.00003-X Published: 2017
Accession Number: WOS:000440395900003
ISBN: 978-0-12-802111-8; 978-0-12-802101-9
https://doi.org/10.1016/B978-0-12-802101-9.00003-X

> "Despite the relative rarity of deceit in clinical research, examples of incorrect results due to bias in trials without blinding (Karlowski et al., 1975) and with single-blind studies reinforce the value of blinding (Henkin et al., 1976)."

Title: **On controversial statistical issues in clinical research**
Author(s): Chow, SC (Chow, Shein-Chung); Song, FY (Song, Fuyu)
Source: OPEN ACCESS JOURNAL OF CLINICAL TRIALS Volume: 7 Pages: 43-51
DOI: 10.2147/OAJCT.S63266 Published: 2015
https://doi.org/10.2147/OAJCT.S63266

> "In randomized and double-blind clinical trials, due to human nature, both patients and the investigator may guess what treatment patients are receiving. Karlowski et al challenged the integrity of the use of randomization and blinding in a randomized, double-blind, placebo-controlled study conducted by the National Institutes of Health (NIH). The study was to evaluate the difference between the prophylactic and therapeutic effects of ascorbic acid for the common cold. After the completion of the study, a questionnaire regarding the knowledge of the treatment assignment was distributed to every subject enrolled in the study (a total of 190 subjects completed the study). Results from the 190 subjects are summarized Table 3.
>
> Table 3 indicates that there is a high percentage of patients who correctly guessed the treatment assignment they received. Thus, there is a reasonable doubt that the blindness may not be preserved during the study. Thus, "How to test for the integrity of blinding in clinical trials?" is an interesting question."
>
> **When Chow in 2015 referred to the Karlowski trial, he knew that the trial was erroneously analyzed since he had previously replied to the letter to the editor by Hemilä in 2006, which pointed out the flaws in the Karlowski trial:**
>
> **Flaws in the Karlowski trial analysis were pointed out by Hemilä:**
> https://pubmed.ncbi.nlm.nih.gov/16572386/
> https://doi.org/10.1002/sim.2347
>
> Response by Chow in 2006 indicating that Chow had read about the problems of the Karlowski trial:
> https://doi.org/10.1002/sim.2348

Title: **The risk of unblinding was infrequently and incompletely reported in 300 randomized clinical trial publications**
Author(s): Bello, S (Bello, Segun); Moustgaard, H (Moustgaard, Helene); Hrobjartsson, A (Hrobjartsson, Asbjorn)
Source: JOURNAL OF CLINICAL EPIDEMIOLOGY Volume: 67 Issue: 10 Pages: 1059-1069
DOI: 10.1016/j.jclinepi.2014.05.007 Published: OCT 2014
PubMed ID: 24973822
https://doi.org/10.1016/j.jclinepi.2014.05.007

> "Blinding procedures may not be effective, and loss of blinding, that is, unblinding, occurs in an unknown proportion of trials. Compromised blinding has generated some concern [3,11-16],"
> [11=Karlowski]
>
> **Flaws in the Karlowski trial analysis were pointed out by Hemilä:**
> https://pubmed.ncbi.nlm.nih.gov/26071891/
> https://doi.org/10.1016/j.jclinepi.2015.05.012
>
> Response by Bello
> https://doi.org/10.1016/j.jclinepi.2015.05.011

Title: **Unequal allocation and allocation concealment**
Author(s): Palys, KE (Palys, Kaitlin E.); Berger, VW (Berger, Vance W.); Grant, WC (Grant, William C.)
Source: STATISTICS IN MEDICINE Volume: 31 Issue: 29 Pages: 4135-4136
DOI: 10.1002/sim.5432 Published: DEC 20 2012
PubMed ID: 23175158
https://doi.org/10.1002/sim.5432

> "It is clear that a study planned as masked can be known with certainty to have become unmasked, and this issue has arisen in some well publicized trials (for example, a 1975 trial of vitamin C [3])"
> [3=Karlowski]

Title: **Some Controversial Issues in Clinical Trials**
Author(s): Chow, SC (Chow, Shein-Chung); Yang, LY (Yang, Lan-Yan); Lu, Y (Lu, Ying)
Source: DRUG INFORMATION JOURNAL Volume: 45 Issue: 2 Pages: 163-174
DOI: 10.1177/009286151104500211 Published: MAR 2011
https://doi.org/10.1177/009286151104500211

> "For illustration purposes, consider the example described in Karlowski et al. A double blind placebo-controlled study was conducted by the National Institutes of Health to evaluate the difference between the prophylactic and therapeutic effects of ascorbic acid for the common cold. At the completion of the study, a questionnaire was distributed to every subject enrolled in the study so that they could guess which treatment they received. Results from the 190 subjects who completed the study are summarized in Table 3."
>
> **When Chow in 2011 referred to the Karlowski trial, he knew that the trial was erroneously analyzed since he had previously replied to the letter to the editor by Hemilä in 2006, which pointed out the flaws in the Karlowski trial:**
>
> **Flaws in the Karlowski trial analysis were pointed out by Hemilä:**
> https://pubmed.ncbi.nlm.nih.gov/16572386/
> https://doi.org/10.1002/sim.2347
>
> Response by Chow in 2006 indicating that Chow had read about the problems of the Karlowski trial:
> https://doi.org/10.1002/sim.2348

Title: **Blindness**
Author(s): Friedman, LM (Friedman, Lawrence M.); Furberg, CD (Furberg, Curt D.); DeMets, DL (DeMets, David L.)
Book Author(s): Friedmen, LM (Friedmen, LM); Furberg, CD (Furberg, CD); DeMets, DL (DeMets, DL)
Source: **FUNDAMENTALS OF CLINICAL TRIALS, FOURTH EDITION Pages: 119-132**
DOI: 10.1007/978-1-4419-1586-3_7 Published: 2010
https://doi.org/10.1007/978-1-4419-1586-3_7

> "A trial of the possible benefits of ascorbic acid in the common cold started out as a double-blind study [6,7]. However, it soon became apparent that many of the participants, most of whom were medical staff, discovered whether they were on ascorbic acid or placebo… Among those participants who claimed not to know the identity of the treatment, ascorbic acid showed no benefit over placebo. In contrast, among participants who knew or suspected what they were on, ascorbic acid did better than placebo. Therefore preconceived notions about the benefit of a treatment, coupled with a subjective response variable, may have yielded biased reporting."
> (p 120)
> [6=Karlowski]
> [7=Lewis 1975]

Title: **Reporting and Interpreting of Results**
Author(s): Friedman, LM (Friedman, Lawrence M.); Furberg, CD (Furberg, Curt D.); DeMets, DL (DeMets, David L.)
Book Author(s): Friedmen, LM (Friedmen, LM); Furberg, CD (Furberg, CD); DeMets, DL (DeMets, DL)
Source: **FUNDAMENTALS OF CLINICAL TRIALS, FOURTH EDITION Pages: 411-425**
DOI: 10.1007/978-1-4419-1586-3_19 Published: 2010
https://doi.org/10.1007/978-1-4419-1586-3_19

> "An evaluation such as that provided by Karlowski and colleagues for a trial of vitamin C is commendable"
> (p 418)

When Furberg in 2010 referred to the Karlowski trial, he knew that the trial was erroneously analyzed since he had previously replied to the letter to the editor by Hemilä in 2008, which pointed out the flaws in the Karlowski trial:

Flaws in the Karlowski trial analysis were pointed out by Hemilä:
https://pubmed.ncbi.nlm.nih.gov/18466312/
https://doi.org/10.1111/j.1538-7836.2008.03006.x

Response by Furberg and Soliman, indicating that Furberg had read about the problems of the Karlowski trial before the update of the textbook:
https://doi.org/10.1111/j.1538-7836.2008.03008.x

Title: **CONSORT 2010 Explanation and Elaboration: updated guidelines for reporting parallel group randomised trials**
Author(s): Moher, D (Moher, David); Hopewell, S (Hopewell, Sally); Schulz, KF (Schulz, Kenneth F.); Montori, V (Montori, Victor); Gotzsche, PC (Gotzsche, Peter C.); Devereaux, PJ (Devereaux, P. J.); Elbourne, D (Elbourne, Diana); Egger, M (Egger, Matthias); Altman, DG (Altman, Douglas G.)
Source: BMJ-BRITISH MEDICAL JOURNAL Volume: 340 Article Number: c869
DOI: 10.1136/bmj.c869 Published: MAR 23 2010
PubMed ID: 22036893
https://doi.org/10.1136/bmj.c869

> "Unblinded outcome adjudicators may differentially assess subjective outcomes, and unblinded data analysts may introduce bias through the choice of analytical strategies, such as the selection of favourable time points or outcomes, and by decisions to remove patients from the analyses. These biases have been well documented.[71,153,159-162]"
> [161=Karlowski]

Title: **Complementary and alternative medicine: Herbs, phytochemicals and vitamins and their immunologic effects**
Author(s): Mainardi, T (Mainardi, Timothy); Kapoor, S (Kapoor, Simi); Bielory, L (Bielory, Leonard)
Source: JOURNAL OF ALLERGY AND CLINICAL IMMUNOLOGY Volume: 123 Issue: 2 Pages: 283-294
DOI: 10.1016/j.jaci.2008.12.023 Published: FEB 2009
PubMed ID: 19203652
https://doi.org/10.1016/j.jaci.2008.12.023

> "The implication that vitamin C is an important mediator of the immune response with an effect on ameliorating the common cold is an idea that stretches back decades, although early studies [36,37] never demonstrated an effect on the duration or intensity of the common cold inpatients supplemented with vitamin C."
> [36=Karlowski]
> [37=Dykes-Meier 1975]
>
> **Flaws in the Karlowski trial analysis were pointed out by Hemilä:**
> http://dx.doi.org/10.1016/j.jaci.2009.06.015 https://helda.helsinki.fi/handle/10138/228310

Title: **Double-blindness protects scientific validity**
Author(s): Furberg, CD (Furberg, C. D.); Soliman, EZ (Soliman, E. Z.)
Source: JOURNAL OF THROMBOSIS AND HAEMOSTASIS Volume: 6 Issue: 2 Pages: 230-231
DOI: 10.1111/j.1538-7836.2007.02836.x Published: FEB 2008
PubMed ID: 18021306
https://doi.org/10.1111/j.1538-7836.2007.02836.x

> "Knowing the intervention assignment can influence, unconsciously or consciously, a participant#s reporting of symptomatic improvement and occurrence of adverse events. In a double-blind, placebo-controlled clinical trial of vitamin C for the treatment and prevention of the common cold, a sizable proportion of the blinded participants, mostly medical staff, broke the blind [2,3]. At the trial conclusion, all participants were asked whether they knew the identity of the blinded intervention that they were on. Interestingly, vitamin C had no benefit as compared to placebo for the common cold among those who said that they did not know which intervention they received. In contrast, vitamin C did significantly better than placebo among those participants who knew the identity of their treatment. This case illustrates the role that preconceived notions can have on the reported benefit of an intervention. Thus, whenever possible, study participants should be blinded to their assigned study medication."
> [2=Karlowski]
> [3=Lewis 1975]
>
> **Flaws in the Karlowski trial analysis were pointed out by Hemilä:**
> https://doi.org/10.1111/j.1538-7836.2008.03006.x
>
> Response by Furberg and Soliman:
> https://doi.org/10.1111/j.1538-7836.2008.03008.x

Title: **Treatment of naturally acquired common colds with zinc: A structured review**
Author(s): Caruso, TJ (Caruso, Thomas J.); Prober, CG (Prober, Charles G.); Gwaltney, JM (Gwaltney, Jack M., Jr.)
Source: CLINICAL INFECTIOUS DISEASES Volume: 45 Issue: 5 Pages: 569-574
DOI: 10.1086/520031 Published: SEP 1 2007
PubMed ID: 17682990
https://doi.org/10.1086/520031

> "The placebo effect in the treatment of colds was first shown >70 years ago [42]
> and has since been demonstrated in subsequent studies [43–45]."
> [44=Chalmers 1975]
> [45=Karlowski]
>
> **When Caruso and Gwaltney in 2007 referred to the Chalmers review and Karlowski trial, they knew that those papers were flawed since they had previously replied to the letter to the editor by Hemilä in 2005, which pointed out the flaws in the two papers:**
>
> "The Chalmers review [4] was shown to be erroneous a decade ago; it has data inconsistent with the original study publications, errors in calculations, and other problems [5, 6]. The particular trial referred to by Chalmers [4] was undertaken by Karlowski et al. [7]… "
> https://pubmed.ncbi.nlm.nih.gov/16080106/
> https://doi.org/10.1086/432629
>
> Response by Caruso and Gwaltney indicating that they had read the text above.
> https://doi.org/10.1086/432628

Title: **Clinical attrition due to biased preclinical assessments of potential efficacy**
Author(s): Lindner, MD (Lindner, Mark D.)
Source: PHARMACOLOGY & THERAPEUTICS Volume: 115 Issue: 1 Pages: 148-175
DOI: 10.1016/j.pharmthera.2007.05.002 Published: JUL 2007
PubMed ID: 17574680
https://doi.org/10.1016/j.pharmthera.2007.05.002

> "Patients have broken blinds by chewing and tasting their capsules (Karlowski et al., 1975; Miller et al., 1977),"

Title: **Large Clinical Trials and Registries-Clinical Research Institutes**
Author(s): Califf, RM (Califf, Robert M.)
Edited by: Gallin JI; Ognibene FP
Source: PRINCIPLES AND PRACTICE OF CLINICAL RESEARCH, 2ND EDITION Pages: 237-263
DOI: 10.1016/B978-012369440-9/50022-0 Published: 2007
https://doi.org/10.1016/B978-012369440-9/50022-0

> "Despite the rarity of deceit in clinical research, examples of incorrect results
> due to bias in trials without blinding[23] and with single-blind studies
> reinforce the value of blinding.[24]"
> [23=Karlowski]

Title: **Bias in clinical intervention research**
Author(s): Gluud, LL (Gluud, LL)
Source: AMERICAN JOURNAL OF EPIDEMIOLOGY Volume: 163 Issue: 6 Pages: 493-501
DOI: 10.1093/aje/kwj069 Published: MAR 15 2006
PubMed ID: 16443796
https://doi.org/10.1093/aje/kwj069

> "If interventions are compared with no intervention, an identical placebo may be used. The compared interventions must be identical in taste, smell, appearance, and mode of administration. Any difference may destroy the blinding (58–60)."
> [59=Karlowski]
> [60=Chalmers 1975]
>
> **Flaws in the Karlowski trial analysis were pointed out by Hemilä:**
> https://pubmed.ncbi.nlm.nih.gov/17426041/
> https://doi.org/10.1093/aje/kwm081
>
> also:
> https://doi.org/10.1093/aje/kwm374

Title: **Double blind, you are the weakest link - goodbye!**
Author(s): Devereaux, PJ (Devereaux, PJ); Bhandari, M (Bhandari, M); Montori, VM (Montori, VM); Manns, BJ (Manns, BJ); Ghali, WA (Ghali, WA); Guyatt, GH (Guyatt, GH)
Source: EQUINE VETERINARY JOURNAL Volume: 37 Issue: 6 Pages: 557-558
DOI: 10.2746/042516405775314916 Published: NOV 2005
PubMed ID: 16295935
https://doi.org/10.2746/042516405775314916

> "Case reports document individual examples of the biases described above (Karlowski et al. 1975 ..."

Title: **Pre-trial evaluation of the potential for unblinding in drug trials: A prototype example**
Author(s): Walter, SD (Walter, SD); Awasthi, S (Awasthi, S); Jeyaseelan, L (Jeyaseelan, L)
Source: CONTEMPORARY CLINICAL TRIALS Volume: 26 Issue: 4 Pages: 459-468
DOI: 10.1016/j.cct.2005.02.006 Published: AUG 2005
PubMed ID: 16054578
https://doi.org/10.1016/j.cct.2005.02.006

> "Some studies have shown that the conclusions differ if subjects whose treatment group has been correctly guessed are eliminated from the analysis [11–15]."
> [15=Karlowski]

Title: **Is acupuncture analgesia an expectancy effect? Preliminary evidence based on participants' perceived assignments in two placebo-controlled trials**
Author(s): Bausell, RB (Bausell, RB); Lao, LX (Lao, LX); Bergman, S (Bergman, S); Lee, WL (Lee, WL); Berman, BM (Berman, BM)
Source: EVALUATION & THE HEALTH PROFESSIONS Volume: 28 Issue: 1 Pages: 9-26
DOI: 10.1177/0163278704273081 Published: MAR 2005
PubMed ID: 15677384
https://doi.org/10.1177/0163278704273081

> "Although the availability of veridical controls is obviously essential in all experimental research, a growing body of evidence suggests that (a) patients may be more difficult to blind in placebo-controlled randomized clinical trials (RCTs) than originally believed (...) and (b) even within this gold-standard experimental context, patients' expectations of the efficacy of a treatment are more predictive of actual outcomes within active treatment groups than within placebo controls (Karlowski et al., 1975; Kirsch&Rosadino, 1993)."

Title: **Changes in beliefs identify unblinding in randomized controlled trials: a method to meet CONSORT guidelines**
Author(s): Rees, JR (Rees, JR); Wade, TJ (Wade, TJ); Levy, DA (Levy, DA); Colford, JM (Colford, JM); Hilton, JF (Hilton, JF)
Source: CONTEMPORARY CLINICAL TRIALS Volume: 26 Issue: 1 Pages: 25-37
DOI: 10.1016/j.cct.2004.11.020 Published: FEB 2005
PubMed ID: 15837450
https://doi.org/10.1016/j.cct.2004.11.020

> "Unblinding may also result from participants' attempts to identify their intervention [13–15]"
> [15=Karlowski]

Title: **Allocation concealment and blinding: when ignorance is bliss**
Author(s): Forder, PM (Forder, PM); Gebski, VJ (Gebski, VJ); Keech, AC (Keech, AC)
Source: MEDICAL JOURNAL OF AUSTRALIA Volume: 182 Issue: 2 Pages: 87-89
DOI: 10.5694/j.1326-5377.2005.tb06584.x Published: JAN 17 2005
PubMed ID: 15651970
https://doi.org/10.5694/j.1326-5377.2005.tb06584.x

> "Patients' or investigators' preconceptions about the value of the treatment may affect a trial's results. For example, in a trial of the effect of vitamin C on symptoms of colds, volunteers took vitamin C or a placebo for 9 months, with an increase in the dose at the onset of a cold.[12] Because of the differences in taste between the vitamin and the placebo, some of the participants became aware of their treatment. In this group, the vitamin C had a reported benefit, but vitamin treatment did not appear to help those who remained blinded. The breakdown of the blinding led to inconclusive results, illustrating the importance of ensuring that blinding is done with care."
> [12=Karlowski]
>
> **Flaws in the Karlowski trial analysis were pointed out by Hemilä:**
> https://pubmed.ncbi.nlm.nih.gov/16175687/
> https://doi.org/10.5694/j.1326-5377.2005.tb06975.x
> https://helda.helsinki.fi/handle/10138/225889

Title: **Analysis of clinical data with breached blindness**
Author(s): Chow, SC (Chow, SC); Shao, J (Shao, J)
Source: STATISTICS IN MEDICINE Volume: 23 Issue: 8 Pages: 1185-1193
DOI: 10.1002/sim.1694 Published: APR 30 2004
PubMed ID: 15083477
https://doi.org/10.1002/sim.1694

> "The first example is a 1-year double-blind placebo-controlled study conducted by the National Institutes of Health to evaluate the difference between the prophylactic and therapeutic effects of ascorbic acid for the common cold (see Reference [3]). A two-group parallel design was used. At the completion of the study, a questionnaire was distributed to everyone enrolled in the study so that they could guess which treatment they had been taking. Results from the 190 subjects who completed the study are given in Table I."
> [3=Karlowski]
>
> **Flaws in the Karlowski trial analysis were pointed out by Hemilä:**
> https://pubmed.ncbi.nlm.nih.gov/16572386/
> https://doi.org/10.1002/sim.2347
>
> Response by Chow
> https://doi.org/10.1002/sim.2348

Title: **Assessment of blinding in clinical trials**
Author(s): Bang, HJ (Bang, HJ); Ni, LY (Ni, LY); Davis, CE (Davis, CE)
Source: CONTROLLED CLINICAL TRIALS Volume: 25 Issue: 2 Pages: 143-156
DOI: 10.1016/j.cct.2003.10.016 Published: APR 2004
PubMed ID: 15020033
https://doi.org/10.1016/j.cct.2003.10.016

> "On the other hand, in a well known trial of vitamin C, the perceptions affected the endpoint concerning cold symptoms [16]."
> [16=Karlowski]
>
> **Flaws in the Karlowski trial analysis were pointed out by Hemilä:**
> https://pubmed.ncbi.nlm.nih.gov/15951244/
> https://doi.org/10.1016/j.cct.2005.04.002
>
> Response by Bang
> https://doi.org/10.1016/j.cct.2005.04.003

Title: **Practical aspects of randomization and blinding in randomized clinical trials**
Author(s): Bridgman, S (Bridgman, S); Dainty, K (Dainty, K); Kirkley, A (Kirkley, A); Maffulli, N (Maffulli, N)
Source: ARTHROSCOPY-THE JOURNAL OF ARTHROSCOPIC AND RELATED SURGERY Volume: 19 Issue: 9 Pages: 1000-1006 DOI: 10.1016/j.arthro.2003.09.023 Published: NOV 2003
PubMed ID: 14608321
https://doi.org/10.1016/j.arthro.2003.09.023

> "Despite the rarity of deceit in clinical research, examples of incorrect results owing to bias in trials without blinding[10] and with single blind studies[11] reinforce the value of this methodology."
> [10=Karlowski]

Title: **Evaluating preference effects in partially unblinded, randomized clinical trials**
Author(s): Halpern, SD (Halpern, SD)
Source: JOURNAL OF CLINICAL EPIDEMIOLOGY Volume: 56 Issue: 2 Pages: 109-115
DOI: 10.1016/S0895-4356(02)00598-X Published: FEB 2003
PubMed ID: 12654404
https://doi.org/10.1016/S0895-4356(02)00598-X

> "However, at least three factors threaten the ability of this traditional, additive model of RCTs to disentangle a treatment's specific effect from the exogenous, or nonphysiologic effects of treatment, including contextual and psychologic effects. First, participant blinding is often difficult to maintain [4–13]. If trials are not blinded, or if they are imperfectly blinded, then between-group differences in treatment adherence, drop-out, cointervention use, symptom reporting, or psychosomatic responses may bias the results."
> [4=Karlowski]

Title: **The reporting of methodological factors in randomized controlled trials and the association with a journal policy to promote adherence to the Consolidated Standards of Reporting Trials (CONSORT) checklist**
Author(s): Devereaux, PJ (Devereaux, PJ); Manns, BJ (Manns, BJ); Ghali, WA (Ghali, WA); Quan, H (Quan, H); Guyatt, GH (Guyatt, GH)
Source: CONTROLLED CLINICAL TRIALS Volume: 23 Issue: 4 Pages: 380-388
DOI: 10.1016/S0197-2456(02)00214-3 Published: AUG 2002
PubMed ID: 12161081
https://doi.org/10.1016/S0197-2456(02)00214-3

> "Unblinded participants and health-care providers may present biased reporting of symptoms, consume or administer powerful cointerventions, and demonstrate differential willingness to continue in a study [1,16]."
> [16=Karlowski]

Title: **Statistical issues - significantly important in medical research**
Author(s): Gellerstedt, M (Gellerstedt, M)
Source: ALLERGY Volume: 57 Issue: 2 Pages: 76-82
DOI: 10.1034/j.1398-9995.2002.1r151.x Published: FEB 2002
PubMed ID: 11929408
https://doi.org/10.1034/j.1398-9995.2002.1r151.x

> "It is also well known that patients can experience benefits just by knowing or believing that they are on active treatment. This effect is also known as the placebo effect (31). One illustration is a study where the effect of ascorbic acid on the common cold was being investigated (32). The treatment showed a positive effect, but it was revealed that some of the subjects had opened the capsules and tasted the contents and thus became aware of receiving active drug or placebo. An analysis for taking into account the 'broken blindness' was performed and showed that there was no effect by this treatment."
> [32=Karlowski]

Title: **On the prevention of the common cold: No help from vitamin C**
Author(s): Spiers, PS (Spiers, PS)
Source: EPIDEMIOLOGY Volume: 13 Issue: 1 Pages: 4-5
DOI: 10.1097/00001648-200201000-00002 Published: JAN 2002
PubMed ID: 11805579
https://doi.org/10.1097/00001648-200201000-00002

> "In a clinical trial by Karlowski et al, the average duration of colds in subjects who guessed correctly that they had been given a placebo throughout the study was 8.6 days. In subjects who guessed correctly that they had been given both prophylactic and therapeutic ascorbic acid, cold duration was only 4.8 days."

Title: **Physician interpretations and textbook definitions of blinding terminology in randomized controlled trials**
Author(s): Devereaux, PJ (Devereaux, PJ); Manns, BJ (Manns, BJ); Ghali, WA (Ghali, WA); Quan, H (Quan, H); Lacchetti, C (Lacchetti, C); Montori, VM (Montori, VM); Bhandari, M (Bhandari, M); Guyatt, GH (Guyatt, GH)
Source: JAMA-JOURNAL OF THE AMERICAN MEDICAL ASSOCIATION Volume: 285 Issue: 15 Pages: 2000-2003
DOI: 10.1001/jama.285.15.2000 Published: APR 18 2001
PubMed ID: 11308438
https://dx.doi.org/10.1001/jama.285.15.2000

> "When unblinded, clinicians may differentially administer treatments other than those under study, influence a patient's compliance with study medication or willingness to continue in the study, and affect patient reporting of symptoms. [1,33]"
> [33=Karlowski]

Title: **How to assess new treatments**
Author(s): Slinger, R (Slinger, R); Moher, D (Moher, D)
Source: WESTERN JOURNAL OF MEDICINE Volume: 174 Issue: 3 Pages: 182-186
DOI: 10.1136/ewjm.174.3.182 Published: MAR 2001
PubMed ID: 11238353
https://www.ncbi.nlm.nih.gov/pmc/articles/PMC1071310/

> "Following allocation to treatment, participants, investigators, or ideally both (that is, double blinding) should be unaware of group assignment because subjective outcomes such as reporting of symptoms and adverse events may be influenced by a knowledge of assignment. For example, some participants in a trial of the use of vitamin C versus placebo for the common cold became aware of their treatment. Vitamin C was found to be beneficial in those who were aware that they were in the treatment arm but not in those who did not know their group assignment.[10]"
> [10=Karlowski]

Title: **Detecting selection bias in randomized clinical trials**
Author(s): Berger, VW (Berger, VW); Exner, DV (Exner, DV)
Source: CONTROLLED CLINICAL TRIALS Volume: 20 Issue: 4 Pages: 319-327
DOI: 10.1016/S0197-2456(99)00014-8 Published: AUG 1999
PubMed ID: 10440559
https://doi.org/10.1016/S0197-2456(99)00014-8

> "the unmasking of a previous treatment code (e.g., owing to tell-tale adverse events [10], emergencies requiring unmasking [11], differences in taste or other distinguishing features between treatments [12],"
> [12=Karlowski]

Title: **Statistics and ethics in medical research**
Author(s): DeMets, DL (DeMets, DL)
Source: SCIENCE AND ENGINEERING ETHICS Volume: 5 Issue: 1 Pages: 97-117
DOI: 10.1007/s11948-999-0059-9 Published: JAN 1999
PubMed ID: 11658016
https://doi.org/10.1007/s11948-999-0059-9

> "A second study illustrates patient bias.[27] Vitamin C had been claimed to be the cure for the common cold. Investigators at NIH decided to conduct an experiment to test this hypothesis. NIH employees with a cold were randomized in a double-blind experiment to receive either placebo or vitamin C. Duration of cold symptoms was the outcome measure. Since the patients in the study were NIH employees, they had access to analytical methods to determine the content of the tablets. At the end of the experiment, the patients were asked if they had unblinded themselves.
>
> Relying on their scientific integrity, their answers were recorded along with their duration of cold symptoms. When the data were analyzed, no differences in duration of cold were observed between treatment groups. However, if the data were stratified by blinding status, those patients who had unblinded themselves and were on placebo had a longer duration of cold symptoms than those who had unblinded themselves and were on vitamin C. For those who had remained blinded, there was no difference in duration of symptoms. Apparently belief about treatment effect among the scientists who were the trial subjects led to substantial bias in the outcome."
> [27=Karlowski]

Title: **Guidelines for quality assurance in multicenter trials: A position paper**
Author(s): Knatterud, GL (Knatterud, GL); Rockhold, FW (Rockhold, FW); George, SL (George, SL); Barton, FB (Barton, FB); Davis, CE (Davis, CE); Fairweather, WR (Fairweather, WR); Honohan, T (Honohan, T); Mowery, R (Mowery, R); O'Neill, R (O'Neill, R)
Source: CONTROLLED CLINICAL TRIALS Volume: 19 Issue: 5 Pages: 477-493
DOI: 10.1016/S0197-2456(98)00033-6 Published: OCT 1998
PubMed ID: 9741868
https://doi.org/10.1016/S0197-2456(98)00033-6

> "Masking may not always succeed because of obvious side effects or laboratory tests. A classic example is the study of the effect of vitamin C on hospital staff members, some of whom analyzed the tablets they received to determine whether they were vitamin C or placebo. Those who had their study medication analyzed reported a shorter duration of symptoms if they were on vitamin C than if they were on placebo. Overall, this study showed no differences in treatment [22]. If all the subjects had had their medication analyzed, they would have jeopardized the trial's validity."
> [22=Karlowski]

Title: **Distinctions between fraud, bias, errors, misunderstanding, and incompetence**
Author(s): DeMets, DL (DeMets, DL)
Source: CONTROLLED CLINICAL TRIALS Volume: 18 Issue: 6 Pages: 637-650
DOI: 10.1016/S0197-2456(97)00010-X Published: DEC 1997
PubMed ID: 9408726
https://doi.org/10.1016/S0197-2456(97)00010-X

> "Patients who agree to be entered into clinical trials are well-motivated and often somewhat knowledgeable about the disease and the available therapies. In their enthusiasm or commitment to the trial, patients can allow biases to enter into their responses. An example of this can be seen in a trial conducted at the National Institutes of Health (NIH) [9] on the effectiveness of vitamin C in the treatment of the common cold. The outcome was duration of cold symptoms. The trial was a randomized, double-blind, placebo-controlled study. Since patients in the study were employees of the NIH, they had either direct or indirect access to laboratories, and were easily able to break the double blind. Overall, there was no discernible difference in the duration of the symptoms between placebo- and vitamin C-treated patients. Patients were asked if they had, in fact, used their own resources in the laboratories to break the blind. For those who had not, vitamin C showed no benefit. For those who had broken the blind, the vitamin C-treated patients reported cold symptoms present for an average of 3.8 fewer days than those who knew they were on the placebo. Since the bias was applied to the primary outcome, it is clear that bias in this case could have created an artificial treatment benefit if all patients had been unblinded."
> [9=Karlowski]

Title: **Double-blindness procedures, rater blindness, and ratings of outcome - Observations from a controlled trial**
Author(s): Basoglu, M (Basoglu, M); Marks, I (Marks, I); Livanou, M (Livanou, M); Swinson, R (Swinson, R)
Source: ARCHIVES OF GENERAL PSYCHIATRY Volume: 54 Issue: 8 Pages: 744-748 Published: AUG 1997
PubMed ID: 9283510
https://doi.org/10.1001/archpsyc.1997.01830200078011

> "Problems in maintaining assessor and/or patient blindness have been long known to researchers. Such problems have been experienced in drug trials involving medical conditions such as the common cold [1]."
> [1=Karlowski]

Title: **Effectiveness of clemastine fumarate for treatment of rhinorrhea and sneezing associated with the common cold**
Author(s): Turner, RB (Turner, RB); Sperber, SJ (Sperber, SJ); Sorrentino, JV (Sorrentino, JV); OConnor, RR (OConnor, RR); Rogers, J (Rogers, J); Batouli, AR (Batouli, AR); Gwaltney, JM (Gwaltney, JM)
Source: CLINICAL INFECTIOUS DISEASES Volume: 25 Issue: 4 Pages: 824-830 DOI: 10.1086/515546 Published: OCT 1997
PubMed ID: 9356796
https://doi.org/10.1086/515546

> "The subjective assessment of symptoms of these mild and self-limited illnesses is readily biased when subjects are unblinded by an inadequate placebo control[28, 29]"
> [28=Karlowski]

Title: **CMAJ endorses the CONSORT statement**
Author(s): Huston, P (Huston, P); Hoey, J (Hoey, J)
Source: CANADIAN MEDICAL ASSOCIATION JOURNAL Volume: 155 Issue: 9 Pages: 1277-1279
Published: NOV 1 1996
PubMed ID: 8911294
https://www.ncbi.nlm.nih.gov/pmc/articles/PMC1335069/

> "Table 1: Making (Blinding): Describe mechanism (e.g., capsules, tablets); similarity of treatment characteristics (e.g., appearance, taste); allocation schedule control (location of code during trial ans when broken); and evidence of successful blinding among participants, person doing intervention, outcome assessors and data analysts.[17,18]"
> [18=Karlowski]

Title: **Statistical analysis of possible bias of clinical judgements due to observing an on-therapy marker variable**
Author(s): Boateng, F (Boateng, F); Sampson, A (Sampson, A); Schwab, B (Schwab, B)
Source: STATISTICS IN MEDICINE Volume: 15 Issue: 16 Pages: 1747-1755 Published: AUG 30 1996
PubMed ID: 8870157
https://doi.org/10.1002/(SICI)1097-0258(19960830)15:16%3C1747::AID-SIM342%3E3.0.CO;2-V

> "Karlowski et al. presented a study designed as double-blind, in which subjects received either vitamin C or lactose (placebo) for nine months, during which time the incidence of colds was monitored. During the study, some subjects indicated that they were biting into and tasting the preparation that they had been given. As a result, the investigators asked all the subjects at the conclusion of the study to guess their assignment group. The results indicated that there was a connection between the subjects' suspicions of the treatment group assigned and the actual treatment they received. This then raises concern regarding the validity of the comparison of vitamin C to placebo."

Title: **Improving the quality of reporting of randomized controlled trials - The CONSORT statement**
Author(s): Begg, C (Begg, C); Cho, M (Cho, M); Eastwood, S (Eastwood, S); Horton, R (Horton, R); Moher, D (Moher, D); Olkin, I (Olkin, I); Pitkin, R (Pitkin, R); Rennie, D (Rennie, D); Schulz, KF (Schulz, KF); Simel, D (Simel, D); Stroup, DF (Stroup, DF)
Source: JAMA-JOURNAL OF THE AMERICAN MEDICAL ASSOCIATION Volume: 276 Issue: 8 Pages: 637-639
DOI: 10.1001/jama.276.8.637 Published: AUG 28 1996
PubMed ID: 8773637
https://doi.org/10.1001/jama.1996.03540080059030
https://www.researchgate.net/publication/14428451_Improving_the_Quality_of_Reporting_of_Randomized_Controlled_Trials_The_CONSORT_Statement

> "Describe mechanism (eg, capsules, tablets); similarity of treatment characteristics (eg, appearance, taste); allocation schedule control (location of code during trial and when broken); and evidence for successful blinding among participants, person doing intervention, outcome assessors, and data analysts.[19,20]"
> [20=Karlowski]

Title: **Blinding during data analysis and writing of manuscripts**
Author(s): Gotzsche, PC (Gotzsche, PC)
Source: CONTROLLED CLINICAL TRIALS Volume: 17 Issue: 4 Pages: 285-290
DOI: 10.1016/0197-2456(95)00263-4 Published: AUG 1996
PubMed ID: 8889343
https://doi.org/10.1016/0197-2456(95)00263-4

> "In a placebo-controlled study of the effect of ascorbic acid for the common cold, the effect disappeared when persons who had guessed they received ascorbic acid because of its taste were excluded from analysis [2]."
> [2=Karlowski]

Title: **Blinding and exclusions after allocation in randomised controlled trials: Survey of published parallel group trials in obstetrics and gynaecology**
Author(s): Schulz, KF (Schulz, KF); Grimes, DA (Grimes, DA); Altman, DG (Altman, DG); Hayes, RJ (Hayes, RJ)
Source: BRITISH MEDICAL JOURNAL Volume: 312 Issue: 7033 Pages: 742-744 Published: MAR 23 1996
PubMed ID: 8605459
https://doi.org/10.1136/bmj.312.7033.742
https://www.ncbi.nlm.nih.gov/pmc/articles/PMC2350472

> "Though investigators have reported compromised blinding,[22,23]
> such candid reporting seems rare."
> [22=Karlowski]

Title: **ASSESSING THE QUALITY OF RANDOMIZED CONTROLLED TRIALS - AN ANNOTATED-BIBLIOGRAPHY OF SCALES AND CHECKLISTS**
Author(s): MOHER, D (MOHER, D); JADAD, AR (JADAD, AR); NICHOL, G (NICHOL, G); PENMAN, M (PENMAN, M); TUGWELL, P (TUGWELL, P); WALSH, S (WALSH, S)
Source: CONTROLLED CLINICAL TRIALS Volume: 16 Issue: 1 Pages: 62-73
DOI: 10.1016/0197-2456(94)00031-W Published: FEB 1995
Accession Number: WOS:A1995QQ51500006
PubMed ID: 7743790
https://doi.org/10.1016/0197-2456(94)00031-W

> "From each scale the following items were recorded: the name of the scale or its principal author, whether the scale was developed to assess the quality of any trial or specific trials (e.g., contrast media, pain), whether quality was defined, the type of quality assessed (i.e., methodological quality or the quality of reporting), how the items were selected, whether the scale included items on four content areas bearing on the internal validity of a trial (patient assignment [35], masking [36]…"
> [36=Karlowski]

Title: **A PROPOSAL FOR STRUCTURED REPORTING OF RANDOMIZED CONTROLLED TRIALS**
Author(s): ANDREW, E (ANDREW, E); ANIS, A (ANIS, A); CHALMERS, T (CHALMERS, T); CHO, M (CHO, M); CLARKE, M (CLARKE, M); FELSON, D (FELSON, D); GOTZSCHE, P (GOTZSCHE, P); GREENE, R (GREENE, R); JADAD, A (JADAD, A); JONAS, W (JONAS, W); KLASSEN, T (KLASSEN, T); KNIPSCHILD, P (KNIPSCHILD, P); LAUPACIS, A (LAUPACIS, A); MEINERT, CL (MEINERT, CL); MOHER, D (MOHER, D); NICHOL, G (NICHOL, G); OXMAN, A (OXMAN, A); PENMAN, MF (PENMAN, MF); POCOCK, S (POCOCK, S); REISCH, J (REISCH, J); SACKETT, D (SACKETT, D); SCHULZ, K (SCHULZ, K); SNIDER, J (SNIDER, J); TUGWELL, P (TUGWELL, P); TYSON, J (TYSON, J); VARIN, F (VARIN, F); WALOP, W (WALOP, W); WALSH, S (WALSH, S); WELLS, G (WELLS, G)
Source: JAMA-JOURNAL OF THE AMERICAN MEDICAL ASSOCIATION Volume: 272 Issue: 24 Pages: 1926-1931
DOI: 10.1001/jama.1994.03520240054041 Published: DEC 28 1994
PubMed ID: 7990245
https://doi.org/10.1001/jama.1994.03520240054041
https://www.jameslindlibrary.org/standards-of-reporting-trials-group-1994/

> Many trial reports do not provide detailed information as to how masking was carried out,[7] whether single, double, or triple, and evidence indicates that masking can affect estimates of intervention effects on subjective outcomes.[12,13,25,26]"
> [26=Karlowski]

Title: **CIMETIDINE SUSPENSION AS ADJUVANT TO ENERGY RESTRICTED DIET IN TREATING OBESITY**
Author(s): RASMUSSEN, MH (RASMUSSEN, MH); ANDERSEN, T (ANDERSEN, T);
BREUM, L (BREUM, L); GOTZSCHE, PC (GOTZSCHE, PC); HILSTED, J (HILSTED, J)
Source: BRITISH MEDICAL JOURNAL Volume: 306 Issue: 6885 Pages: 1093-1096
DOI: 10.1136/bmj.306.6885.1093 Published: APR 24 1993
PubMed ID: 8388286
https://dx.doi.org/10.1136/bmj.306.6885.1093
https://www.ncbi.nlm.nih.gov/pmc/articles/PMC1677486

> "In contrast to recommendations,[9,10] however, the effectiveness of the blinding has only rarely been tested and related to outcome. In a placebo controlled study of the effect of vitamin C on the common cold, an apparent dose related effect on the duration of symptoms was noted, but the effect disappeared when subjects identifying the vitamin by its special taste were excluded [11]."
> [11=Karlowski]

Title: **METAANALYSIS OF 2ND-LINE ANTIRHEUMATIC DRUGS - SAMPLE-SIZE BIAS AND UNCERTAIN BENEFIT**
Author(s): GOTZSCHE, PC (GOTZSCHE, PC); PODENPHANT, J (PODENPHANT, J); OLESEN, M (OLESEN, M); HALBERG, P (HALBERG, P)
Source: JOURNAL OF CLINICAL EPIDEMIOLOGY Volume: 45 Issue: 6 Pages: 587-594
DOI: 10.1016/0895-4356(92)90130-F Published: JUN 1992
PubMed ID: 1535101
https://dx.doi.org/10.1016/0895-4356(92)90130-F

> "Most trials are small, however, and since several drugs have conspicuous side effects, bias may be introduced by loss of blinding [2].
> [2=Karlowski]"

Title: **A SURVEY OF PHARMACISTS RECOMMENDATIONS FOR FOOD SUPPLEMENTS IN THE USA AND UK**
Author(s): NELSON, MV (NELSON, MV); BAILIE, G (BAILIE, G)
Source: JOURNAL OF CLINICAL PHARMACY AND THERAPEUTICS Volume: 15 Issue: 2 Pages: 131-139
DOI: 10.1111/j.1365-2710.1990.tb00367.x Published: APR 1990
PubMed ID: 2341491
https://doi.org/10.1111/j.1365-2710.1990.tb00367.x

> "The belief that vitamin C will prevent or shorten the duration of the common cold is widespread although not substantiated (18)."
> [18=Karlowski]

Title: **METHODOLOGY AND OVERT AND HIDDEN BIAS IN REPORTS OF 196 DOUBLE-BLIND TRIALS OF NONSTEROIDAL ANTIINFLAMMATORY DRUGS IN RHEUMATOID-ARTHRITIS**
Author(s): GOTZSCHE, PC (GOTZSCHE, PC)
Source: CONTROLLED CLINICAL TRIALS Volume: 10 Issue: 1 Pages: 31-56
DOI: 10.1016/0197-2456(89)90017-2 Published: MAR 1989
PubMed ID: 2702836
https://dx.doi.org/10.1016/0197-2456(89)90017-2

> "Randomization and blinding are probably the two most important safeguards against bias, but details of the methods are often lacking [33-36,38]. These labels may be used too freely, for example, trials called double-blind are not always double-blind [41] or may become unblinded [42,43]."
> [42=Karlowski]

Title: **CHEMOTHERAPY OF RHINOVIRUS COLDS**
Author(s): SPERBER, SJ (SPERBER, SJ); HAYDEN, FG (HAYDEN, FG)
Source: ANTIMICROBIAL AGENTS AND CHEMOTHERAPY Volume: 32 Issue: 4 Pages: 409-419
DOI: 10.1128/AAC.32.4.409 Published: APR 1988
PubMed ID: 2897829
https://doi.org/10.1128/AAC.32.4.409
https://www.ncbi.nlm.nih.gov/pmc/articles/PMC172192/

> "One clinical trial of ascorbic acid showed that the apparent benefit in the vitamin C recipients was accounted for by volunteers who had tasted the contents of their capsules and correctly identified the treatment. Reanalysis with omission of these subjects found no evidence of a treatment benefit [14,61]"
> [14=Chalmers 1975]
> [61=Karlowski]

Title: **RESEARCH METHODS IN NUTRITION AND DIETETICS - DESIGN, DATA-ANALYSIS, AND PRESENTATION**
Author(s): MONSEN, ER (MONSEN, ER); CHENEY, CL (CHENEY, CL)
Source: JOURNAL OF THE AMERICAN DIETETIC ASSOCIATION Volume: 88 Issue: 9 Pages: 1047-1065
Published: SEP 1988
PubMed ID: 3047199
https://doi.org/10.1016/S0002-8223(21)07952-9

> "Example. The National Institutes of Health conducted a double-blind (neither subject nor investigator was informed as to which treatment group the subject was assigned) randomized controlled trial of the effectiveness of ascorbic acid on reducing the frequency and severity of the common cold (32). A lactose capsule placebo that could be easily distinguished from the vitamin C tablet by taste was used, although the investigators gave little thought to the possibility that their subjects might actually bite into the capsules.
>
> Early in the study, the investigators learned that their volunteers were quite curious and many had bitten into the capsules; a significant number of subjects knew which medication they were receiving. Although the study was no longer a double-blind study, it did illustrate an association between severity and duration of symptoms and knowledge of the medication taken. Among those subjects who tasted their capsules, those receiving vitamin C had shorter, milder colds, while the converse was true for the placebo group. Among those subjects who remained blind to their treatment, no effect of vitamin C was seen."
> [32=Karlowski]

Title: **THE PROBLEMS OF TASTE IN PLACEBO MATCHING - AN EVALUATION OF ZINC GLUCONATE FOR THE COMMON COLD**
Author(s): FARR, BM (FARR, BM); GWALTNEY, JM (GWALTNEY, JM)
Source: JOURNAL OF CHRONIC DISEASES Volume: 40 Issue: 9 Pages: 875-879
DOI: 10.1016/0021-9681(87)90187-1 Published: 1987
PubMed ID: 3298301
https://doi.org/10.1016/0021-9681(87)90187-1

> "One very important trial by Karlowski et al. showed that the trend toward milder symptoms in the ascorbic acid group was accounted for by volunteers who had tasted the contents of their capsules and correctly guessed their medication. Reanalysis omitting the subjects who had "unblinded" themselves by tasting their capsules resulted in no difference in the severity or duration of colds between the ascorbic acid and placebo groups."

Title: **THE APPLICATION OF CLINICAL TOXICOLOGY**
Author(s): BUTTERWORTH, KR (BUTTERWORTH, KR); MANGHAM, BA (MANGHAM, BA)
Source: CRC CRITICAL REVIEWS IN TOXICOLOGY Volume: 18 Issue: 2 Pages: 81-128
DOI: 10.3109/10408448709089857 Published: 1987
PubMed ID: 3311643
https://doi.org/10.3109/10408448709089857

> "Rarely do publications of the results of trials discuss possible inadequate matching. An exception is the vitamin C study [108,109] which suffered from a breakdown of the double-blind system. One possible reason given by the investigators was that, in the rush to begin the study, the contents of the capsules were not carefully produced. The lactose placebo could easily be distinguished from ascorbic acid by taste, as the study subjects quickly discovered."
> [108=Karlowski]
> [109=Lewis 1975]

Title: **ASCORBIC-ACID**
Author(s): TRUSWELL, AS (TRUSWELL, AS)
Source: NEW ENGLAND JOURNAL OF MEDICINE Volume: 315 Issue: 11 Pages: 709-709 Published: SEP 11 1986
https://doi.org/10.1056/NEJM198609113151113

> "In another five combined trials there appeared to be slight amelioration of symptoms, which was not statistically significant [10,13,15,16,26]."
> [10=Karlowski]
>
> **In fact, all listed five trials found statistically significant benefit on some outcome, see Table II in:**
> http://dx.doi.org/10.1016/S0899-9007(96)00223-7
> https://helda.helsinki.fi/handle/10138/225877
> http://www.mv.helsinki.fi/home/hemila/H/HH_1996_NUT.pdf
>
> **see Table 21 (p 45) in:**
> http://hdl.handle.net/10138/20335

Title: **ASSESSMENT OF DOUBLE-BLINDNESS AT THE CONCLUSION OF THE BETA-BLOCKER HEART ATTACK TRIAL**
Author(s): BYINGTON, RP (BYINGTON, RP); CURB, JD (CURB, JD); MATTSON, ME (MATTSON, ME)
Source: JAMA-JOURNAL OF THE AMERICAN MEDICAL ASSOCIATION Volume: 253 Issue: 12 Pages: 1733-1736
DOI: 10.1001/jama.253.12.1733 Published: 1985
PubMed ID: 3974051
https://doi.org/10.1001/jama.1985.03350360059018

> Only a few double-blinded clinical trials report the success or failure of blinding. If reported at all, the assessment of blindness is usually noted in three or four sentences in the primary report of the trial [8-10].
> [9=Karlowski]

Title: **BLINDNESS AND THE VALIDITY OF THE DOUBLE-BLIND PROCEDURE**
Author(s): HUGHES, JR (HUGHES, JR); KRAHN, D (KRAHN, D)
Source: JOURNAL OF CLINICAL PSYCHOPHARMACOLOGY Volume: 5 Issue: 3 Pages: 138-142 Published: 1985
PubMed ID: 3998203
https://pubmed.ncbi.nlm.nih.gov/3998203
https://journals.lww.com/psychopharmacology/Abstract/1985/06000/Blindness_and_the_Validity_of_the_Double_Blind.3.aspx

> "Although several of these studies further hypothesized that failure to maintain blindness threatened the validity of their results [4,5,9,10], only one study actually tested this hypothesis.
>
> … The only study that provided such a demonstration tested ascorbic acid as a prophylactic and supplemental treatment of the common cold [9]. In this study, the effect of supplemental ascorbic acid on many cold symptoms was greater among subjects who "knew" their drug assignment than among subjects who "did not know" their drug assignment. In addition, supplemental ascorbic acid reduced duration in "unblinded" subjects but not in "blinded" subjects [9,10]. Thus, unlike the present study, this study indicated that failure to maintain blindness did affect the magnitude of the drug effects"
> [9=Karlowski]
> [10=Lewis 1975]

Title: **SCIENTIFIC CHALLENGES IN THE APPLICATION OF RANDOMIZED TRIALS**
Author(s): KRAMER, MS (KRAMER, MS); SHAPIRO, SH (SHAPIRO, SH)
Source: JAMA-JOURNAL OF THE AMERICAN MEDICAL ASSOCIATION Volume: 252 Issue: 19 Pages: 2739-2745
DOI: 10.1001/jama.252.19.2739 Published: 1984
PubMed ID: 6492351
https://doi.org/10.1001/jama.1984.03350190041017

> "Bias should be suspected whenever differences in outcome appear only in subjects who are unblinded. This can occur if, among active treatment recipients, those who correctly identify their treatment have better outcomes than those who either are unsure or believe they received the active treatment. It can also occur if unblinded subjects receiving the placebo have worse outcomes than placebo recipients who remained blind. A good example of the use of this bias assessment strategy was one of the RCTs of vitamin C in the prevention and treatment of the common cold [19]. The shorter duration and lesser severity of cold experienced by the vitamin C group were confined to subjects who were unblinded; in those who remained blind, no such differences were found."
> [19=Karlowski]

Title: **THE DOUBLE-BLIND IN DANGER - UNTOWARD CONSEQUENCES OF INFORMED CONSENT**
Author(s): BROWNELL, KD (BROWNELL, KD); STUNKARD, AJ (STUNKARD, AJ)
Source: AMERICAN JOURNAL OF PSYCHIATRY Volume: 139 Issue: 11 Pages: 1487-1489 Published: 1982
PubMed ID: 6753613
https://doi.org/10.1176/ajp.139.11.1487

> "In a study of the prophylactic use of ascorbic acid for the common cold, Karlowski and associates found that correct estimates of drug assignment outnumbered incorrect estimates by a ratio of 3.5:1 by patients receiving placebo as well as by those receiving ascorbic acid."

Title: **HOW BLIND WAS THE PATIENT BLIND IN AMIS**
Author(s): HOWARD, J (HOWARD, J); WHITTEMORE, AS (WHITTEMORE, AS); HOOVER, JJ (HOOVER, JJ); PANOS, M (PANOS, M)
Source: CLINICAL PHARMACOLOGY & THERAPEUTICS Volume: 32 Issue: 5 Pages: 543-553
DOI: 10.1038/clpt.1982.201 Published: 1982
PubMed ID: 7127995
https://doi.org/10.1038/clpt.1982.201

> "In one well-known case a trial of vitamin C was presumably compromised by the subjects' efforts to unblind their assignments [8]. Their perceptions may well have affected the endpoint data concerning cold symptoms."
> [8=Karlowski]

Title: **A POTPOURRI OF RCT TOPICS**
Author(s): CHALMERS, TC (CHALMERS, TC); FRIEDEWALD, WT (FRIEDEWALD, WT); MEINERT, CL (MEINERT, CL); FISHER, LD (FISHER, LD); ELASHOFF (ELASHOFF); GEHAN, EA (GEHAN, EA); KLIMT (KLIMT); STAMLER (STAMLER); SMITH (SMITH); BUCHWALD, H (BUCHWALD, H); ROCKETTE, HE (ROCKETTE, HE)
Source: CONTROLLED CLINICAL TRIALS Volume: 3 Issue: 3 Pages: 285-298
DOI: 10.1016/0197-2456(82)90012-5 Published: 1982
PubMed ID: 7151441
https://doi.org/10.1016/0197-2456(82)90012-5

> "DR. CHALMERS: I am not going to argue with you about the reason. Another factorial study we did was an evaluation of ascorbic acid for both the prevention and treatment of the common cold [12]. We had a group getting no ascorbic acid either for treatment or for prevention; we had a group getting it only for prevention; a group getting it only for treatment; and a group getting three grams a day for prevention who got three more grams a day if they got a cold. It worked beautifully. We answered both questions simultaneously, except for the fact that some volunteers cheated and we had to stop the study because the placebo was detected and those receiving it dropped out more often than those on ascorbic acid."
> [12=Karlowski]

Title: **PROTECTING THE SCIENTIFIC INTEGRITY OF A CLINICAL-TRIAL - SOME ETHICAL DILEMMAS**
Author(s): HOWARD, J (HOWARD, J); FRIEDMAN, L (FRIEDMAN, L)
Source: CLINICAL PHARMACOLOGY & THERAPEUTICS Volume: 29 Issue: 5 Pages: 561-569
DOI: 10.1038/clpt.1981.78 Published: 1981
PubMed ID: 7214785
https://doi.org/10.1038/clpt.1981.78

> "Physicians are in the habit of making informed guesses in caring for patients, and they will not necessarily refrain from doing so simply because they are supposed to be blind. They may in fact deliberately try to break the blind for the fun of it [20] or to gain more knowledge about their patients."
> [20=Karlowski]

Title: **A METHOD FOR ASSESSING THE QUALITY OF A RANDOMIZED CONTROL TRIAL**
Author(s): CHALMERS, TC (CHALMERS, TC); SMITH, H (SMITH, H); BLACKBURN, B (BLACKBURN, B); SILVERMAN, B (SILVERMAN, B); SCHROEDER, B (SCHROEDER, B); REITMAN, D (REITMAN, D); AMBROZ, A (AMBROZ, A)
Source: CONTROLLED CLINICAL TRIALS Volume: 2 Issue: 1 Pages: 31-49
DOI: 10.1016/0197-2456(81)90056-8 Published: 1981
PubMed ID: 7261638
https://doi.org/10.1016/0197-2456(81)90056-8

> "Testing of Blinding:
> It is not sufficient to assume that a double-bhnd procedure is effective. In good studies the physicians and their patients are quizzed at the end of the study to determine whether or not they have guessed the medication involved. The data may be important in interpretation of the results [3]"
> [3=Karlowski]

Title: **NUTRITIONAL ASPECTS OF ASCORBIC-ACID - USES AND ABUSES**
Author(s): VILTER, RW (VILTER, RW)
Source: WESTERN JOURNAL OF MEDICINE Volume: 133 Issue: 6 Pages: 485-492 Published: 1980
PubMed ID: 7008359
https://www.ncbi.nlm.nih.gov/pmc/articles/PMC1272392

> In 1975, Dykes and Meier [73] found no evidence to support claims of clinically important efficacy, and Chalmers and co-workers,[74,75] after running a controlled experiment themselves on the prophylactic effect of vitamin C against the common cold, considered only 8 of 15 reports scientifically acceptable, and these 8 were not convincing."
> [74=Karlowski]
> [75=Chalmers 1975]

Title: **PSYCHOLOGICAL PERSPECTIVE FOR DOUBLE-BLIND TRIALS**
Author(s): ZIFFERBLATT, SM (ZIFFERBLATT, SM); WILBUR, CS (WILBUR, CS)
Source: CLINICAL PHARMACOLOGY & THERAPEUTICS Volume: 23 Issue: 1 Pages: 1-10 Published: 1978
PubMed ID: 618704
https://doi.org/10.1002/cpt19782311

> "For example, Karlowski and co-workers detected a high incidence of blind breaking among participants in a double-blind study of ascorbic acid. When knowledge of treatment assignment was included in the analysis, the differences in severity of colds was largely eliminated"

Title: **VITAMIN-C AND COMMON COLD**
Author(s): TAFT, G (TAFT, G); FIELDHOUSE, P (FIELDHOUSE, P)
Source: PUBLIC HEALTH Volume: 92 Issue: 1 Pages: 19-25
DOI: 10.1016/S0033-3506(78)80097-3 Published: 1978
https://doi.org/10.1016/S0033-3506(78)80097-3

> "In March 1975 a double blind study was carried out at the National Institute of Health [15]. Three hundred and eleven employees were randomly assigned to take either 1 g ascorbic acid or placebo three times daily for 9 months. At the onset of a cold an extra 3 g was given. (Certain individuals were excluded from the study including those with histories of renal stones or high blood uric acid levels, the pregnant and those taking anticoagulants.) Results indicated that vitamin C had at best only a minor influence on the duration and severity of colds, and an insignificant effect on their incidence."
> [15=Karlowski]

Title: **VITAMIN-C AND COMMON COLD**
Author(s): [Anonymous] ([Anonymous])
Source: BMJ-BRITISH MEDICAL JOURNAL Volume: 1 Issue: 6010 Pages: 606-607 Published: 1976
https://doi.org/10.1136/bmj.1.6010.606
https://www.ncbi.nlm.nih.gov/pmc/articles/PMC1639003/

> "A more recent American study of adult employees of the National Institutes of Health reported in 1975 found no significant prophylactic or therapeutic benefit from ascorbic acid.[14]"
> [14=Karlowski]

Title: **PERSPECTIVE FROM CONTROLLED INVESTIGATIONS ON CHEMOTHERAPY FOR VIRAL RESPIRATORY-INFECTIONS**
Author(s): JACKSON, GG (JACKSON, GG)
Source: JOURNAL OF INFECTIOUS DISEASES Volume: 133 Pages: A83-A92 Supplement: S Published: 1976
PubMed ID: 778309
https://doi.org/10.1093/infdis/133.supplement_2.a83
http://www.ncbi.nlm.nih.gov/pmc/articles/pmc7110384/
https://www.jstor.org/stable/pdf/30107941.pdf

> "Intermittent doses of as much as 8 g of vitamin C per day at the time of exposure to infection may have reduced the duration of symptomatic illness, but continuous large doses had no beneficial effect in preventing common acute respiratory disease of diverse etiology [35, 36]."
> [36=Karlowski]

Title: **NONSPECIFIC ENHANCERS OF RESISTANCE IN MAN**
Author(s): FLORMAN, AL (FLORMAN, AL); HOLZMAN, RS (HOLZMAN, RS)
Source: JOURNAL OF PEDIATRICS Volume: 87 Issue: 6 Pages: 1094-1102
DOI: 10.1016/S0022-3476(75)80121-1 Published: 1975
PubMed ID: 1102645
https://doi.org/10.1016/S0022-3476(75)80121-1

> “The literature [88-98] describing vitamin C's effect on the common cold has been reviewed in detail by Dykes and Meier [99]. These authors concluded that,
> ‘... no clear reproducible pattern of efficacy has emerged...’
> and that,
> ‘The unrestricted use of ascorbic acid for these purposes cannot be advocated on the basis of the evidence currently available.’ "
> [98=Karlowski]

Listing S2: Statistical calculations for Figure 1

Duration, N, and SE are extracted from [29]

```
> Karlowski <- read.csv("Karlowski.csv")
> Karlowski
  Dose Duration   Change  N   SE    Var InvVar
1    0     7.14   0.0000 65 0.46 0.2116 4.7259
2    3     6.46  -9.5238 56 0.39 0.1521 6.5746
3    3     6.71  -6.0224 52 0.53 0.2809 3.5600
4    6     5.92 -17.0868 76 0.40 0.1600 6.2500
```

Un-weighted linear regression of change in duration by dose:

```
> Karlowski1  <- lm(Karlowski$Change ~ Karlowski$Dose -1)
> summary(Karlowski1)
Call:
lm(formula = Karlowski$Change ~ Karlowski$Dose - 1)

Coefficients:
               Estimate Std. Error t value Pr(>|t|)
Karlowski$Dose  -2.7622     0.2067  -13.36 0.0009


Residual standard error: 1.519 on 3 degrees of freedom
Multiple R-squared:  0.9835, Adjusted R-squared:  0.978
F-statistic: 178.6 on 1 and 3 DF,  p-value: 0.0009057
```

Weight by inverse variance gives more narrow SE for slope:

```
> Karlowski2  <- lm(Karlowski$Change ~ Karlowski$Dose -1, weights =
    Karlowski$InvVar)
> summary(Karlowski2)

Call:
lm(formula = Karlowski$Change ~ Karlowski$Dose - 1, weights =
    Karlowski$InvVar)

Coefficients:
               Estimate Std. Error t value Pr(>|t|)
Karlowski$Dose  -2.8238     0.1741  -16.22  0.00051


Residual standard error: 3.096 on 3 degrees of freedom
Multiple R-squared:  0.9887, Adjusted R-squared:  0.985
F-statistic:   263 on 1 and 3 DF,  p-value: 0.0005101
```

Drawing Figure 1:

```
> #95% CI at 6 grams per day:
> (cilo <- (-2.7622  - 1.96*0.2067)*6)
[1] -19.00399
> (cihi <- (-2.7622  + 1.96*0.2067)*6)
[1] -14.14241
> #6 g:
> (-2.7622*6)
[1] -16.5732
> #12 g:
> (-2.7622*12)
[1] -33.1464
>
> plot(Karlowski$Change ~ Karlowski$Dose,
+      pch =16,
+      cex =2,
+      #xaxs = "i",
+      xlim=c(0,12.2),
+      xaxp  = c(0, 12, 4),
+      ylim=c(-36,1),
+      yaxp  = c(-30, 0, 3),
+      xlab="Vitamin C dose (g/day)",
+      ylab = "Decrease in common cold duration (%)"
+      )
>
> polygon(c(0,6,6),c(0,-14.14241, -19.00399), col ="deepskyblue")
> segments(0, 0, 6, -16.5732, lw=3)
> segments(6, -16.5732, 12, -33.1464, lt=2, lw=3)
> points(Karlowski$Change ~ Karlowski$Dose, pch =16,cex =2)
```

Listing S3: Statistical calculations for Table 5

```
> Coulehan74 <- matrix(c(143,178,93,227),nrow=2)
> Coulehan74
     [,1] [,2]
[1,]  143   93
[2,]  178  227
> fisher.test(Coulehan74)

    Fisher's Exact Test for Count Data

data:  Coulehan74
p-value = 5.79e-05
alternative hypothesis: true odds ratio is not equal to 1
95 percent confidence interval:
 1.39642 2.75656
sample estimates:
odds ratio
   1.95885

> chisq.test(Coulehan74, correct=F)

    Pearson's Chi-squared test

data:  Coulehan74
X-squared = 16.52, df = 1, p-value = 4.81e-05

> # Coulehan (1974)
> riskratio(143, 93, 321, 320)
           Disease Nondisease Total
Exposed        143        178   321
Nonexposed      93        227   320

    Risk ratio estimate and its significance probability

data:  143 93 321 320
p-value = 4.88e-05
95 percent confidence interval:
 1.24221 1.89148
sample estimates:
[1] 1.53284
```

Listing S4: Statistical calculations for Figure 3

```
read.csv("CITRIS.csv")

CITRISsurv <- Surv(CITRIS$Duration, CITRIS$Dead)

CitrisCRQ <- crq(CITRISsurv ~ vitc , data=CITRIS, tau = c(1:15/50), method
    ="PengHuang")

plot(summary(CitrisCRQ, 1:15/50, R = 10000),
    ylim = c(-5, 25),nrow=1,ncol=1,
    xlab="Quantile of survival time",
    ylab ="Extra time alive (days)")
```

Listing S5: Statistical calculations for Figure 4

```
*    COVID A to Z trial
import delimited "C:\Users\hemila\OneDrive\Life\Calculations\COVID.csv",
     encoding(Big5) clear

sqreg duration vitc, quantile(0.05 0.1 0.15 0.2 0.25 0.3 0.35 0.4 0.45 0.5 0.55
     0.6 0.65 0.7 0.75 0.8 0.85 0.9) reps(500)

preserve

gen q = _n*5 in 1/18
list

gen _b_vitc  = .
gen _lb_vitc = .
gen _ub_vitc = .

replace _b_vitc = _b[q05:vitc] in 1
replace _lb_vitc = _b[q05:vitc] - _se[q05:vitc]*invnormal(.975) in 1
replace _ub_vitc = _b[q05:vitc] + _se[q05:vitc]*invnormal(.975) in 1

local i = 2
foreach q of numlist 10(5)90 {
                                                                  replace
     _b_vitc = _b[q`q':vitc] in `i'
        replace _lb_vitc = _b[q`q':vitc] - _se[q`q':vitc]*invnormal(.975) in
     `i'
        replace _ub_vitc = _b[q`q':vitc] + _se[q`q':vitc]*invnormal(.975) in
     `i++'
}

keep q _b_* _lb_* _ub_*
keep in 1/18
reshape long _b_ _lb_ _ub_, i(q) j(var) string
set scheme s1color
twoway rarea _lb_ _ub_ q ,  astyle(ci)  fcolor(midblue) acolor(%50)
     lcolor(black)|| ///
     line _b_ q, lcolor(black) lwidth(0.5)  ///
     yline(0,    lcolor(black) lwidth(0.4) lpattern(dash))  ///
     yline(-1.2,   lcolor(blue)  lwidth(0.4) lpattern(shortdash))  ///
     subtitle("") ///
     yscale(range(-32,1)) ylabel(-16(4)0)   ///
     text( -30 14 "3" -30 34 "5" -30 48 "7" -30 60 "9" -30 78 "11" -30 84 "15"
     -30 90 "28" , color(red))    ///
   text(-27 14 "Duration in the placebo group (days)", color(red) placement(e))
     ///
   text(-3.5 0 "-1.2 day mean", color(blue) placement(e)) ///
   by(var, xrescale note(""))  ///
   legend(order(2 "Effect"  1 "95% CI") rows(2) )   ///
   ytitle(Effect on COVID-19 duration (days))                        ///
   ylab(,angle(0) format(%7.0gc))                              ///
   xlab(0(20)100) xtitle(Percentile of COVID-19 duration)  ///


restore
```

Listing S6: Statistical calculations for Table 6

```
> Kalil
              studlab event.e n.e event.c n.c
1        Fujii (2020)      25 107      21 104
2 Ferron-Celma (2009)       6  10       4  10
3       Fowler (2014)       7  16       5   8
4  Nabil Habib (2017)      12  50      18  50
5       Galley (1997)      11  16       8  14
6    Schneider (2011)       6  29       6  29
>
> Kalil.meta <- metabin(event.e, n.e, event.c, n.c, studlab,
+                           data = Kalil,
+                           method = "MH",
+                           label.e ="vitamin C",
+                           label.c ="Control",
+                           comb.fixed =F,
+                           comb.random =F,
+                           print.CMH = F
+ )
Warning messages:
1: Use argument 'fixed' instead of 'comb.fixed' (deprecated).
2: Use argument 'random' instead of 'comb.random' (deprecated).
> summary(Kalil.meta)
                        RR           95%-CI
Fujii (2020)        1.1571 [0.6924; 1.9338]
Ferron-Celma (2009) 1.5000 [0.6024; 3.7351]
Fowler (2014)       0.7000 [0.3233; 1.5157]
Nabil Habib (2017)  0.6667 [0.3599; 1.2348]
Galley (1997)       1.2031 [0.6864; 2.1088]
Schneider (2011)    1.0000 [0.3650; 2.7394]

Number of studies: k = 6
Number of observations: o = 443
Number of events: e = 129




> (sum(Kalil$n.e))
[1] 228

> (sum(Kalil$n.c))
[1] 215
```
